# Durability of the Effectiveness of Heterologous COVID-19 Vaccine Regimens in Thailand: Retrospective Cohort Study Using National Registration Data

**DOI:** 10.2196/48255

**Published:** 2024-03-05

**Authors:** Ponlagrit Kumwichar, Chittawan Poonsiri, Siobhan Botwright, Natchalaikorn Sirichumroonwit, Bootsakorn Loharjun, Supharerk Thawillarp, Nontawit Cheewaruangroj, Amorn Chokchaisiripakdee, Yot Teerawattananon, Virasakdi Chongsuvivatwong

**Affiliations:** 1 Department of Epidemiology Faculty of Medicine Prince of Songkla University Songkhla Thailand; 2 Health Intervention and Technology Assessment Program Ministry of Public Health Nonthaburi Thailand; 3 Department of Medical Services Institute of Medical Research and Technology Assessment Ministry of Public Health Nonthaburi Thailand; 4 Department of Disease Control Ministry of Public Health Nonthaburi Thailand; 5 Big Data Institute Ministry of Digital Economy and Society Bangkok Thailand

**Keywords:** COVID-19, heterologous vaccine, vaccine, vaccine effectiveness, durability, time, waning, real-world, public health, vaccination strategy, health outcome, vaccines, vaccination, unvaccinated, big data, registry, registries, health outcomes, effectiveness, SARS-CoV-2, cohort, database, databases, vaccinated, Cochran-Mantel-Haenszel, Mantel-Haenszel, regression, risk, risks, association, associations, odds ratio, odds ratios

## Abstract

**Background:**

The durability of heterologous COVID-19 vaccine effectiveness (VE) has been primarily studied in high-income countries, while evaluation of heterologous vaccine policies in low- and middle-income countries remains limited.

**Objective:**

We aimed to evaluate the duration during which the VE of heterologous COVID-19 vaccine regimens in mitigating serious outcomes, specifically severe COVID-19 and death following hospitalization with COVID-19, remains over 50%.

**Methods:**

We formed a dynamic cohort by linking records of Thai citizens aged ≥18 years from citizen vital, COVID-19 vaccine, and COVID-19 cases registry databases between May 2021 and July 2022. Encrypted citizen identification numbers were used to merge the data between the databases. This study focuses on 8 common heterologous vaccine sequences: CoronaVac/ChAdOx1, ChAdOx1/BNT162b2, CoronaVac/CoronaVac/ChAdOx1, CoronaVac/ChAdOx1/ChAdOx1, CoronaVac/ChAdOx1/BNT162b2, BBIBP-CorV/BBIBP-CorV/BNT162b2, ChAdOx1/ChAdOx1/BNT162b2, and ChAdOx1/ChAdOx1/mRNA-1273. Nonimmunized individuals were considered for comparisons. The cohort was stratified according to the vaccination status, age, sex, province location, month of vaccination, and outcome. Data analysis employed logistic regression to determine the VE, accounting for potential confounders and durability over time, with data observed over a follow-up period of 7 months.

**Results:**

This study includes 52,580,841 individuals, with approximately 17,907,215 and 17,190,975 receiving 2- and 3-dose common heterologous vaccines (not mutually exclusive), respectively. The 2-dose heterologous vaccinations offered approximately 50% VE against severe COVID-19 and death following hospitalization with COVID-19 for 2 months; however, the protection significantly declined over time. The 3-dose heterologous vaccinations sustained over 50% VE against both outcomes for at least 8 months, as determined by logistic regression with durability time-interaction modeling. The vaccine sequence consisting of CoronaVac/CoronaVac/ChAdOx1 demonstrated >80% VE against both outcomes, with no evidence of VE waning. The final monthly measured VE of CoronaVac/CoronaVac/ChAdOx1 against severe COVID-19 and death following hospitalization at 7 months after the last dose was 82% (95% CI 80.3%-84%) and 86.3% (95% CI 83.6%-84%), respectively.

**Conclusions:**

In Thailand, within a 7-month observation period, the 2-dose regimens could not maintain a 50% VE against severe and fatal COVID-19 for over 2 months, but all of the 3-dose regimens did. The CoronaVac/CoronaVac/ChAdOx1 regimen showed the best protective effect against severe and fatal COVID-19. The estimated durability of 50% VE for at least 8 months across all 3-dose heterologous COVID-19 vaccine regimens supports the adoption of heterologous prime-boost vaccination strategies, with a primary series of inactivated virus vaccine and boosting with either a viral vector or an mRNA vaccine, to prevent similar pandemics in low- and middle-income countries.

## Introduction

The effectiveness of COVID-19 vaccine is typically determined through randomized controlled trials prior to market approval [1]. Most clinical trials on COVID-19 vaccines focused on homologous vaccination [2], whereas most vaccination programs in low- and middle-income countries (LMICs) use heterologous vaccination for the primary series [3-7]. Recently, evidence has consistently shown that the vaccine effectiveness (VE) of a 3-dose homologous messenger ribonucleic acid (mRNA) vaccine regimen against severe COVID-19 appears to wane at 6 months after the third dose. However, evidence regarding the durability of 3-dose heterologous vaccination regimens remains limited [8-11]. Most research on the durability of heterologous COVID-19 VE have been conducted in high-income countries [12-14]. Although real-world VE studies provide information about the protective effects of heterologous regimens, considerable variations exist across studies because of differences in the vaccine combinations used, unmeasured confounders, and differences in the vaccination eligibility criteria over time and across countries [15]. To our knowledge, no population-based studies have been conducted on the durability of VE of the heterologous prime-boost regimens primarily used in LMICs, which involve a primary series of inactivated vaccines followed by an mRNA or a viral vector vaccine.

In Thailand, a heterologous vaccination strategy was implemented without strong evidence of VE due to supply constraints [16]. The primary series of vaccinations was initially administered using inactivated virus vaccines, followed by viral vector vaccines, whereas mRNA vaccines were mainly used as a booster dose [7,17,18]. Although test-negative case-control studies have been conducted of heterologous vaccination effectiveness in Thailand [7,17,18], these studies are limited in size and follow-up period and therefore were unable to determine the temporal relationship [19]. Thailand is not unique in this regard. To the best of our knowledge, no LMIC has modeled VE durability or waning over time in population-based cohort studies.

Thailand has maintained a national citizen registry linked to national health databases since 1993 [20]. Specialized registries have been established for COVID-19 testing, COVID-19 cases, and COVID-19 vaccination [21]. By linking these records, we examined a dynamic cohort of the Thai population to assess the durability of VE of heterologous vaccine sequences, providing insights for future pandemics. This study aims to evaluate the duration during which the VE of heterologous vaccine sequences against severe COVID-19 and death following hospitalization for COVID-19 (fatal COVID-19) remains over 50%.

## Methods

### Study Design

This was a dynamic cohort study in which participants entered or exited the cohort at different timepoints during the study period, depending on when they met the eligibility criteria for entering or leaving the cohort [22]. The time frame of this study was from May 2021 (when the COVID-19 vaccination started in Thailand) to July 2022 (when the compulsory registration of all COVID-19 cases ended). We assumed that all individuals residing in the same province would have a similar risk of exposure to SARS-CoV-2 during the pandemic.

### Setting

**Figure 1:**
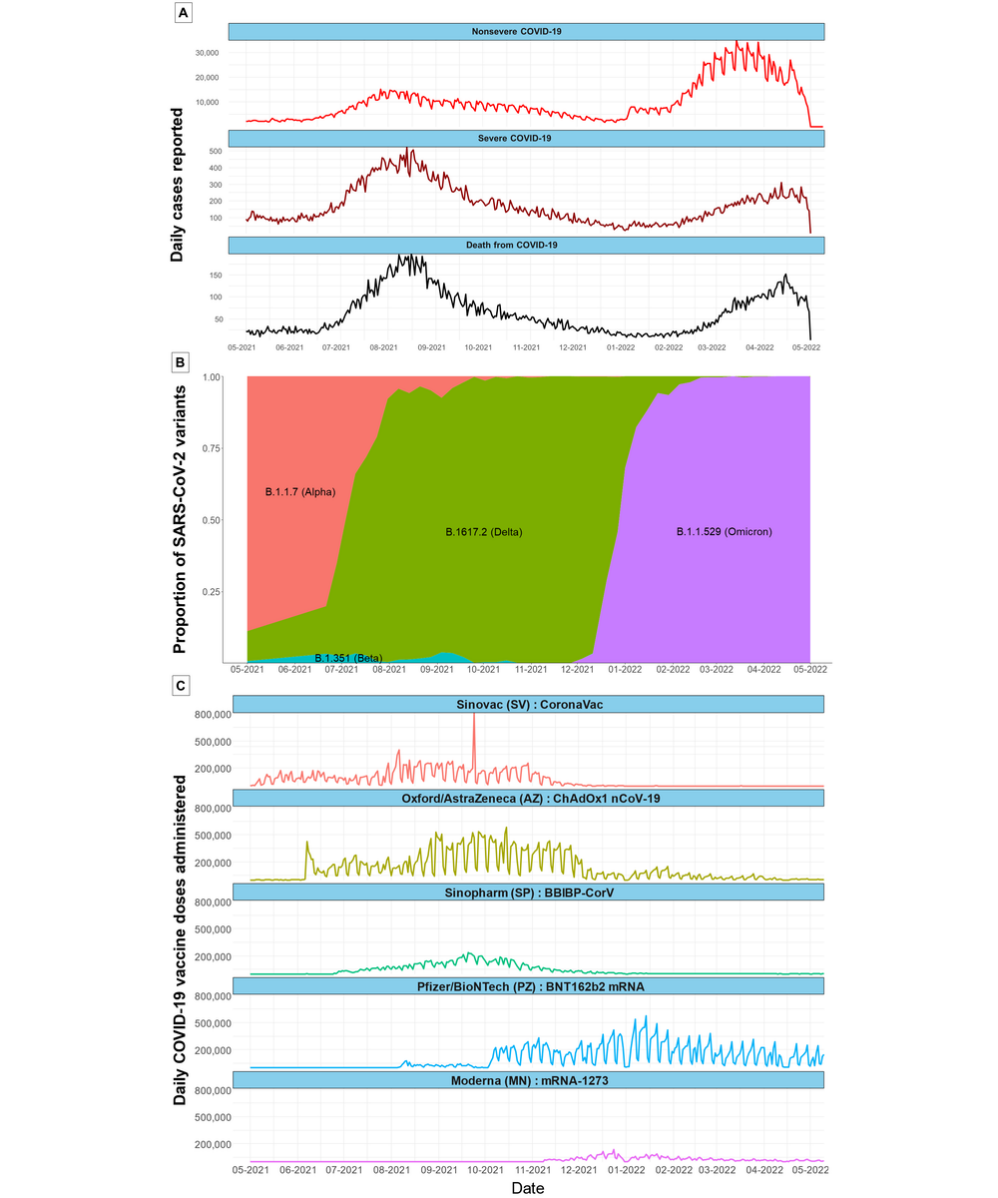
The overall situation of the COVID-19 outbreak during the recruitment period of the dynamic cohort. (A) Daily number of cases of COVID-19 notified (top 2 rows) and COVID-19 deaths (bottom row). (B) SARS-CoV-2 variants circulating in Thailand (pink: Alpha; green: Delta; lilac: Omicron). (C) Daily number of vaccine doses administered by product during the study period.

Figure 1A displays the time series of notified SARS-CoV-2 infection, severe COVID-19, and deaths following hospitalization with COVID-19, while Figure 1B shows the circulating variants. Figure 1A shows that the national trends in COVID-19 case notifications and COVID-19 vaccine administration from May 2021 to June 2022. In Figure 1B, from May 2021 to May 2022, B.1.1.7 (Alpha) was rapidly replaced by B.1617.2 (Delta), which caused a peak in severe and fatal COVID-19 cases. The Delta variant has since been replaced by the B.1.1.529 (Omicron) variant, which has a higher infection rate but reduced severity [23]. A marked reduction was noted in the reporting of COVID-19 cases after the reimbursement protocol for COVID-19 was integrated into the universal health coverage program [24]. Therefore, no data analysis was conducted beyond July 2022. Figure 1C shows that the first vaccine to be rolled out was CoronaVac (Sinovac [SV], Sinovac Biotech), for which the rate of uptake remained steady until the end of 2021. ChAdOx1 nCoV-19 (University of Oxford/AstraZeneca [AZ]) was rolled out in June 2021 and was the main vaccine used during the Delta variant outbreak. Simultaneously, a limited volume of BBIBP-CorV (Sinopharm [SP], National Biotech Group, Beijing Institute of Biological Products) became available via national academies and the private sector. BNT162b2 (Pfizer/BioNTech Inc [PZ]) was introduced at the end of 2021 and was expected to become the main vaccine used in 2022. The market share of mRNA-1273 (Moderna [MN]) has remained low.

### Data Sources

Data were retrieved from national databases of the Thai Ministry of Public Health (MoPH) and the Ministry of Interior. The core data set is the civil registration database, which contains all the citizen identification records. In Thailand, citizens must show their citizen identification cards to access public services such as government-funded health care. COVID-19 vaccine procurement and delivery in both the public and private sectors were monitored by the MoPH through the Mor Prom database, which holds details, including the location of vaccination, citizen identification code, vaccine type, and vaccine batch. Another MoPH database, CO-WARD, recorded all reverse transcription polymerase chain reaction (RT-PCR) test results and clinical outcomes, including severity and death. COVID-19 testing, quarantine, treatment, and vaccination costs were all recorded for reimbursement through national health insurance schemes. In addition, all deaths are recorded by law in a separate national Ministry of Interior (Thailand) database. All the databases were linked using encrypted citizen identification in the analysis.

### Participants

In this study, the participants were all Thai citizens aged 18 years or older because the 2021 vaccination policy only included the adult population. At the start of the study period (May 1, 2021), all participants were nonimmunized, meaning that they had neither received a vaccination nor had a prior SARS-CoV-2 infection. Upon vaccination, participants moved into a group defined by the sequence of vaccines received: SV-AZ, AZ-PZ, SV-SV-AZ, SV-AZ-AZ, SV-AZ-PZ, SP-SP-PZ, AZ-AZ-PZ, and AZ-AZ-MN. Thus, the participant vaccination status could change over time sequentially from nonimmunized to 1, 2, and 3 doses. Participants were excluded from the cohort if they had evidence of SAR-CoV-2 infection (positive antigen test kit [ATK] or RT-PCR) or if they died from any cause. Otherwise, they were followed up until the end of the study period (July 31, 2022).

### Exposures and Outcomes

Table 1 lists the outcomes and exposure variables. Outcomes were defined as untested/uninfected or infected (positive RT-PCR results). The infected group was further classified as nonsevere or severe (oxygen saturation ≤94% on room air or rapid progressive pneumonia based on the doctor’s decision) [25] and fatal infection (any hospitalized death with SARS-CoV-2 infection). The assessment of severity was assessed daily, without missing data [26]. We omitted the evaluation of VE in asymptomatic patients or patients with nonsevere COVID-19 because of underreporting issues. Additionally, our analysis omitted cases of severe COVID-19 that resulted in death prior to hospitalization. This decision was based on our data exploration, which showed that such scenarios accounted for less than 1% of COVID-19–related deaths. On December 1, 2021, the national policy changed such that individuals who tested positive using an ATK were defined as having SARS-CoV-2 infection, whereas previously, the case definition required confirmation using RT-PCR [27]. Owing to this change in the classification of cases during the study period, we included only severe COVID-19 cases and deaths following hospitalization for COVID-19 (fatal COVID-19) as the outcomes of interest. The primary independent variable in this study was the vaccine regimen. Due to the presence of multiple categories of vaccination sequences, our analysis focused on the 8 most prevalent heterologous sequences. Other vaccine sequences administered to an insufficient number of individuals (see Data Availability) were excluded from this report. The control group consisted of nonimmunized individuals. Additional variables for confounder adjustment encompassed age, sex, province of residence, and the calendar month of infection (a proxy for the predominant variant during each period). The results were consolidated based on the time elapsed 2 months following the receipt of the last vaccine dose, as evidence suggests that the decline in the immune response against the SARS-CoV-2 virus starts 3 months after the final dose [28].

**Table 1:** Variables used in the data analysis to determine the durability of heterologous COVID-19 vaccine regimens in Thailand.

Variables			Abbreviation		Categories
**Outcomes**					
	Severe COVID-19	Y_1_		No evidence of SARS-CoV-2 infection or SARS-COV-2 infection without severe COVID-19 (reference) Severe COVID-19	
	Fatal COVID-19	Y_2_		No evidence of SARS-CoV-2 infection or SARS-COV-2 infection without death (reference) Death following hospitalization with COVID-19	
**Exposures**					
	Vaccine sequence	V		No immunization (reference) SV^a^-AZ^b^ AZ-PZ^c^ SV-SV-AZ SV-AZ-AZ SV-AZ-PZ SP-SP^d^-PZ AZ-AZ-PZ AZ-AZ-MN^e^	
**Stratification factors**					
	Age group (years)	x_1_		18-40 (reference) 41-60 41-60 61-80 over 80	
	Sex	x_2_		Female (reference) Male	
	Province of residence	x_3_		Bangkok (capital, reference) Other provinces (76 provinces)	
	Month of infection^f^	x_4_		July 2021 (reference) August 2021-July 2022 (11 months)	
	Time since 2 months after the last dose of each vaccine sequence (months)^g^	T		0 (2 months after the last dose of each vaccine sequence, reference) 1-5	

^a^SV: Sinovac (CoronaVac).

^b^AZ: AstraZeneca (ChAdOx1).

^c^PZ: Pfizer/BioNTech (BNT162b2).

^d^SP: Sinopharm (BBIBP-CorV).

^e^MN: Moderna (mRNA-1273).

^f^Month of infection is a categorical variable.

^g^Time elapsed since 2 months after the last dose of each vaccine sequence was treated as a categorical variable for the Mantel-Haenszel method and as a continuous variable in the logistic regression model (1 month was assumed to be 30 calendar days).

### Data Manipulation Methods

The encrypted citizen identification is used to merge data from each database. Individuals were classified according to their vaccination sequence at the end of each month and further stratified according to sex, age group, province of residence, SARS-CoV-2 infection status, and severity of infection. Once infected (positive ATK or RT-PCR results), the participants were excluded from the analysis for subsequent months.

### Data Analysis

#### Estimation of VE Over Time

The variables employed in our analysis (Table 1) were used in stratified cross-tabulation and logistic regression analyses. The outcome variables were severe COVID-19 (Y1) and death following COVID-19 (Y2). They were analyzed separately and reported. The exposure variable was the vaccine sequence, for which we included two 2-dose and five 3-dose regimens. Other vaccine sequences had small sample sizes and were omitted.

In this study, 2 analytical methods were employed to evaluate VE. The first method, the Mantel-Haenszel risk ratio [29], calculated VE_t_ as VE_t_ = 1 – aRR_t_ [30], where aRR is the adjusted risk ratio, adjusted for other variables (x) and t represents a categorical time variable (2-7 months after the last dose), as indicated by data observed within 7 months since the last dose. In this study, T+2 was used as the measure of t, based on the assumption that VE begins to wane 2 months after the last dose. The second method, logistic regression, assesses VE durability using the formula: VE_t_ = 1 – aOR_t_ [30], where aOR is the adjusted odds ratio, adjusted for other variables (x), and t is a continuous time variable measuring the months after the last dose. This approach allows for projecting aOR to calculate VE_t_ more than 7 months postvaccination. Given the rarity of severe and fatal COVID-19 cases (incidence less than 1 per 1000), logistic regression was used to estimate the aOR of the vaccine, and the aOR was used to estimate the VE. This is based on the assumption that the odds ratio (OR) approximates the relative risk in rare event scenarios [30-32]. The hypothesis posits that VE may either persist or wane over time. Performing both analyses over a 7-month period aims to determine the concordance between the 2 methods, particularly whether aOR aligns with adjusted risk ratio in a rare outcome situation.

#### Stratified Cross-Tabulation and Pooling Effect (Mantel-Haenszel Method)

Each outcome and exposure variable were cross-tabulated using all the stratification factors, as shown in Table 1. Eventually, 51,744 tables (2×2) per month (T) were obtained for each vaccine sequence and outcome. The Mantel-Haenszel relative risk [33] of each specific vaccine sequence was calculated using 2×2 tables. By subtracting the relative risk values of all the vaccine sequences from unity, we determined the effectiveness of the vaccine sequences for each month. All VE points were plotted against the month since the last dose of each vaccine sequence to illustrate the waning of VEs over time.

#### Logistic Regression

The primary objective of the regression analysis was to estimate the time since the last vaccination dose at which VE decreased to 50%—the minimum threshold recommended by the World Health Organization [31]. According to the variables listed in Table 1, the main independent variables of interest were vaccine sequence (V), time since 2 months after the last dose (T), and their interaction (VT). Other independent variables with their categories were all denoted as *x*. Using the abbreviations in Table 1, we constructed the model to predict an individual’s probability of obtaining the outcome using the following formula for each vaccine sequence:




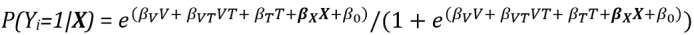




where i indexes the set of outcomes, β_X_ is the coefficient for each category of other variables, and β_0_ denotes the intercept term. From the model, β_V_ would be the natural logarithm (ln) of OR_V_ at T=0. For each incremental month, the ln(OR_V_) would change by β_VT_. Given that VE_i_ is VE against Y_i_ of each vaccine sequence, the VE_i_ for any T is 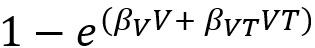. A statistically significant β_VT_ indicates a significant change in the VE_i_ over time. A variance inflation factor of greater than 5 was considered to indicate collinearity among noninteraction variables [34]. The following formula was used to obtain the estimated time at which VE_i_ declined to 50% (T_VE=50%_): T_VE=50%_ = (ln(0.5) – β_V_)/β_VT_. Hence, the number of months after the last dose of each vaccine sequence at which VE_i_ declined to 50% was T_VE=50%_+2. Additionally, a booster dose may be required.

### Subgroup Analysis

We conducted a subgroup analysis by age group, utilizing the logistic regression model to examine the consistency of VE durability for each age group in relation to the overall population, where *x* represents variables other than the age group. All analyses were performed using epiDisplay version 3.5.0.2 [35], car version 3.1 [36], and tidyverse version 1.3.1 [37] packages in R version 4.1.1 (R Core Team 2021; R: A Language and Environment for Statistical Computing, R Foundation for Statistical Computing). *P*<.05 was considered statistically significant.

### Ethics Approval

This study was approved by the human research ethics committee of Songkla University (REC 65-373-18-4). The waiver of consent for accessing encrypted data was granted by the MoPH and Ministry of Interior (Thailand).

## Results

### Participants

A dynamic cohort of 52,580,841 individuals was established throughout the study period, beginning in May 2021. The cohort size decreased over the course of the study, as the participants exited because of SARS-CoV-2 infection (positive ATK or RT-PCR). For the 8 vaccine regimens included in our analysis, by the end of the study, 17,907,215 and 17,190,975 individuals who received 2 and 3 doses, respectively (not mutually exclusive), remained in the cohort. Thus, 45.73% (24,048,933/52,580,841) of the cohort comprised individuals who either received vaccine sequences other than the 8 primary sequences or received no vaccination at all. Table 2 describes the characteristics of individuals receiving each vaccine sequence. The columns are arranged in a chronological order based on the date on which the Thai government policy included the vaccine regimen, starting with the inactivated virus vaccine, followed by the viral vector vaccine, and finally the mRNA vaccine.

**Table 2:** Characteristics of individuals aged ≥18 years who completed heterologous COVID-19 vaccination from May 2021 to May 2022.

Demographics		SV^a^-AZ^b^	AZ-PZ^c^	SV-SV-AZ	SV-AZ-AZ	SV-AZ-PZ	SP^d^-SP-PZ	AZ-AZ-PZ	AZ-AZ-MN^e^	Nonimmunized^f^
Total (N)^g^		15,949,194	1,958,021	2,403,520	1,862,878	4,703,404	1,951,546	5,166,290	1,103,337	44,424,291
**Sex, n (%)**										
	Female	8,414,839 (52.8)	968,649 (49.5)	1,331,232 (55.4)	1,020,155 (54.8)	2,584,869 (55)	1,068,301 (54.7)	2,862,671 (55.4)	635,662 (57.6)	23,267,161 (52.4)
	Male	7,534,355 (47.2)	989,372 (50.5)	1,072,288 (44.6)	842,723 (45.2)	2,118,535 (45)	883,245 (45.3)	2,303,619 (44.6)	467,675 (42.4)	21,157,130 (47.6)
Age (years), mean (SD)		46.2 (16.2)	49.7 (16.9)	42.3 (12.0)	47.1 (16.1)	47.5 (16.1)	39.5 (14.1)	47.2 (16.6)	47.2 (16.6)	46.3 (17.3)
**Age group (years), n (%)**										
	18-40	6,287,541 (39.4)	864,171 (44.1)	1,040,358 (43.3)	681,877 (36.6)	165,3476 (35.2)	1,106,757 (56.7)	1,791,961 (34.7)	454,076 (41.2)	19,669,319 (44.3)
	40-60	6,998,580 (43.9)	752,975 (38.5)	1,270,408 (52.9)	84,7059 (45.5)	2,125,381 (45.2)	713,478 (36.6)	1,895,300 (36.7)	376,090 (34.1)	17,215,748 (38.7)
	60-80	2,502,226 (15.7)	309,146 (15.8)	89,667 (3.7)	314,308 (16.9)	875,393 (18.6)	121,323 (6.2)	1,388,652 (26.9)	256,191 (23.2)	6,970,081 (15.7)
	Over 80	160,847 (1)	31,729 (1.6)	3087 (0.1)	19,634 (1)	49,154 (1)	9988 (0.5)	90,377 (1.7)	16,980 (1.5)	569,143 (1.3)
**Regional area, n (%)**										
	Bangkok (capital)	254,872 (1.6)	86,010 (4.4)	384,195 (16)	38,383 (2.1)	83,496 (1.8)	95,015 (4.9)	1,304,126 (25.2)	358,327 (32.5)	3,675,053 (8.3)
	Central	2,927,216 (18.4)	301,783 (15.4)	546,683 (22.7)	503,522 (27)	832,144 (17.7)	573,932 (29.4)	1,479,139 (28.6)	352,876 (32)	9,954,471 (22.4)
	North	1,885,484 (11.8)	485,370 (24.8)	150,490 (6.3)	295,738 (15.9)	701,329 (14.9)	257,491 (13.2)	306,847 (6)	49,952 (4.5)	4,477,914 (10.1)
	Northeast	6,874,988 (43.1)	697,679 (35.6)	557,002 (23.2)	553,144 (29.7)	2,343,599 (49.8)	552,451 (28.3)	1,100,220 (21.3)	174,410 (15.8)	15,085,451 (34)
	East	1,025,189 (6.4)	110,808 (5.7)	159,441 (6.6)	177,323 (9.5)	231,917 (4.9)	187,462 (9.6)	392,273 (7.6)	80,371 (7.3)	3,226,324 (7.2)
	West	723,149 (4.5)	59,807 (3)	118,212 (4.9)	113,776 (6.1)	118,322 (2.5)	88,224 (4.5)	207,480 (4)	40,709 (3.7)	2,059,025 (4.6)
	South	2,258,296 (14.2)	216,564 (11.1)	487,497 (20.3)	180,992 (9.7)	392,597 (8.4)	196,971 (10.1)	376,205 (7.3)	46,692 (4.2)	5,946,053 (13.4)
**Interval duration between doses (weeks), median (IQR)**										
	Interval between 1st and 2nd doses (weeks)	3.4 (3-4)	5.1 (4-8.6)	3 (3-3.4)	3.4 (3-4)	3.4 (3-4)	3 (3-4)	12 (11.6-12)	12 (11-12)	N/A^h^
	Interval between 2nd and 3rd doses (weeks)	N/A	N/A	16 (14-19)	19 (16-22)	20 (17-25)	16 (12-20)	17 (15-20)	17.1 (15-20)	N/A

^a^SV: Sinovac (CoronaVac).

^b^AZ: AstraZeneca (ChAdOx1).

^c^PZ: Pfizer/BioNTech (BNT162b2).

^d^SP: Sinopharm (BBIBP-CorV).

^e^MN: Moderna (mRNA-1273).

^f^Nonimmunized population (reference) in May 2021.

^g^Individuals who completed vaccination using a different vaccine sequence were not included.

^h^N/A: not applicable.

In May 2021, there were 44,424,291 nonimmunized individuals. This number decreased over time as vaccination campaigns continued. At the end of the study period, this number was reduced to 76,402. The sex distribution across all vaccine types remained relatively balanced. The number of individuals in the 18-40 years and 40-60 years age groups differed across the vaccine regimens. The interval between doses 1 and 2 of the vaccine was approximately within 1 month if dose 1 was SV or SP and 2-3 months if dose 1 was AZ, as per national guidelines [25]. The duration between doses 2 and 3 varied between 1 and 6 months. Amid the emergence of the Omicron variant, PZ and MN were the predominant vaccines used.

### Effectiveness of the Vaccines

**Figure 2:**
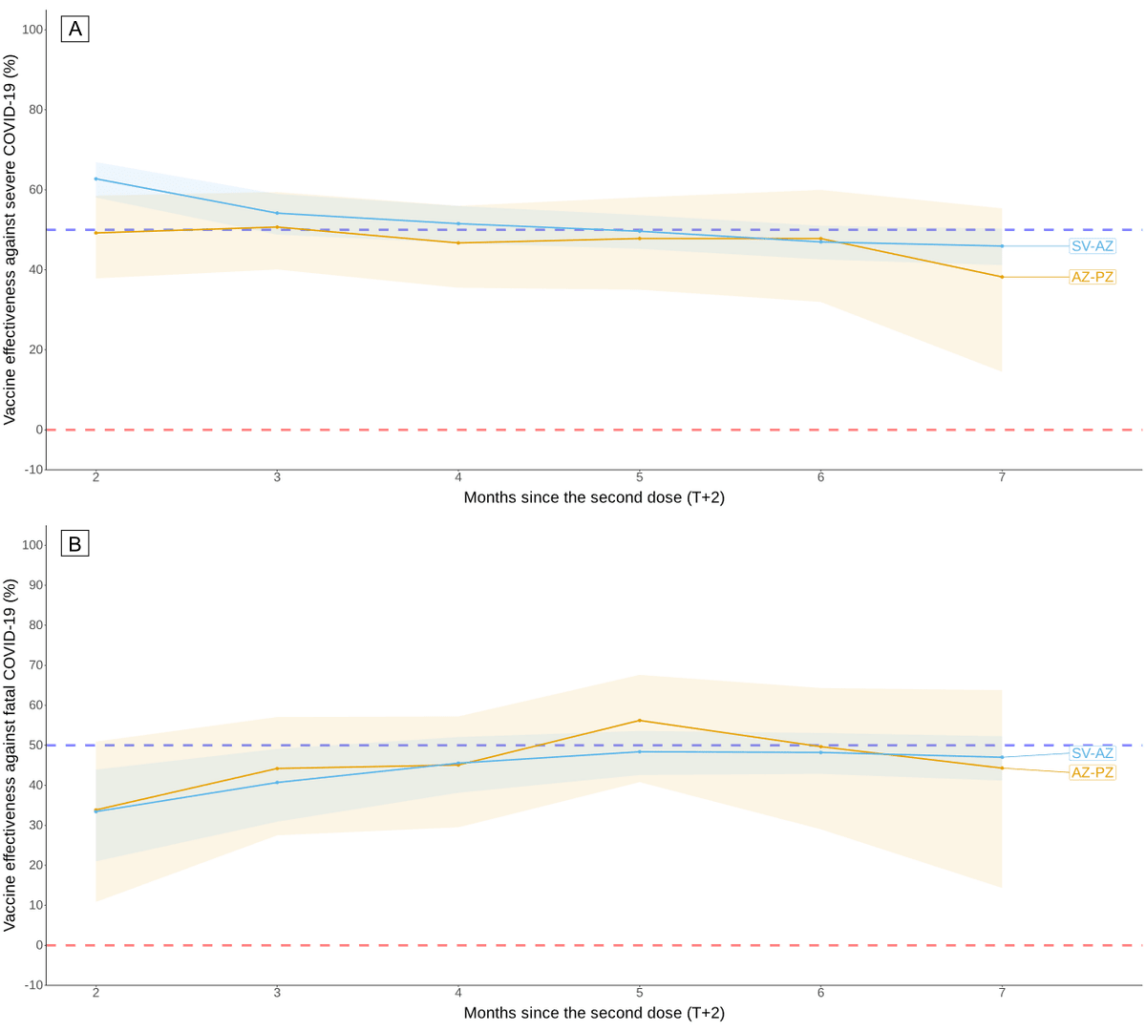
Vaccine effectiveness of 2-dose vaccine sequences at preventing (A) severe and (B) fatal COVID-19 estimated using the Mantel-Haenszel risk ratio method with 95% CI ribbon from July 2021 to July 2022. For each month, the risk ratios of having the outcomes were stratified by the number of months after dose 2 and adjusted by sex, age group, province, and month of infection. AZ: AstraZeneca (ChAdOx1); PZ: Pfizer/BioNTech (BNT162b2); SV: Sinovac (CoronaVac).

**Figure 3:**
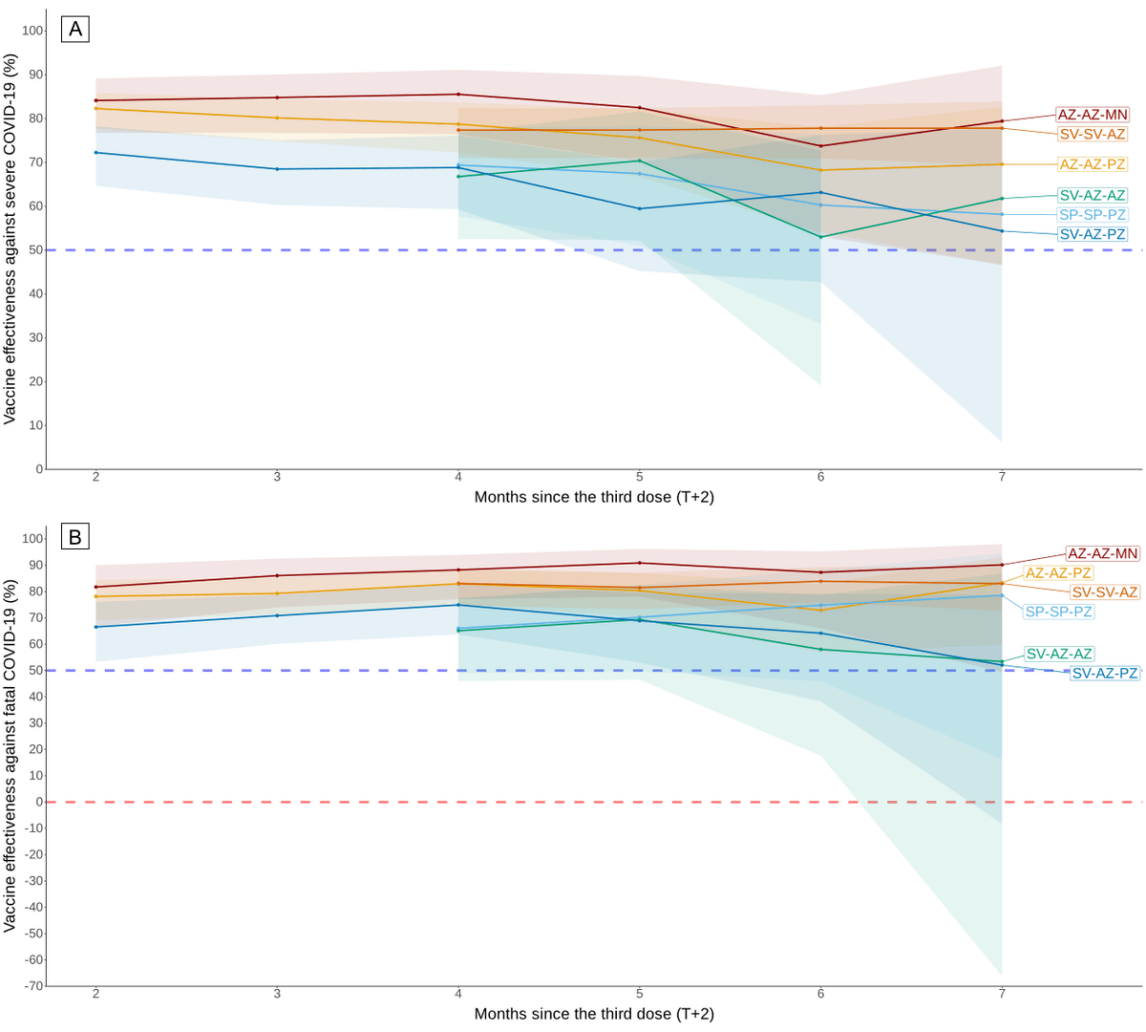
Vaccine effectiveness of 3-dose vaccine sequences at preventing (A) severe and (B) fatal COVID-19 estimated using the Mantel-Haenszel risk ratio method with 95% CI ribbon from July 2021 to July 2022. For each month, the risk ratios of having the outcomes were stratified by the number of months after dose 3 and adjusted by sex, age groups, province, and month of infection. For SV-SV-AZ, SV-AZ-AZ, and SP-SP-PZ vaccine sequences, the vaccine effectiveness at 2 and 3 months after dose 3 was excluded due to an insufficient number of outcomes, resulting in CIs with an infinite range. AZ: AstraZeneca (ChAdOx1); MN: Moderna (mRNA-1273); PZ: Pfizer/BioNTech (BNT162b2); SP: Sinopharm (BBIBP-CorV); SV: Sinovac (CoronaVac).

The effectiveness of each heterologous vaccine regimen over time is shown in Figure 2, Figure 3, and Table 3. The estimated VEs over time from the logistic regression are illustrated in Figures S1-S2 of Multimedia Appendix 1. Table S1 of Multimedia Appendix 1 shows no significant collinearity (variance inflation factor<5) among the noninteraction variables in all logistic regression models. The 2-dose heterologous vaccination provided approximately 50% of the initial protection against severe and fatal COVID-19. After dose 2, protection against severe COVID-19 declined to less than 50% effectiveness at 5.9 months for SV-AZ and 4.7 months for AZ-PZ. The protective effect against death following COVID-19 was too low to compute a T_VE=50%_+2.

All 3-dose heterologous vaccine sequences provided better protection than 2-dose heterologous vaccinations. SV-SV-AZ and SV-AZ-AZ provided persistent protection against both outcomes by over 80% and 60%, respectively, from the initial period to 7 months after dose 3 without evidence of waning. There was no statistical significance of the waning of VE against death between the SP-SP-PZ and AZ-AZ-MN groups. The T_VE_+2=50% protective effect against severe and fatal COVID-19 for each other 3-dose heterologous vaccine sequence varied from 8.7 to 14.6 months. Figures 2 and 3 demonstrate the concordance in estimating VE using logistic regression within 7 months following the last dose for all vaccine sequences, as illustrated in Figures S1-S2 of Multimedia Appendix 1.

**Table 3:** Vaccine effectiveness of each vaccine combination at 2 and 7 months after the last dose of vaccine.

Vaccine sequence				VE^a^ at 2 months after the last dose (95% CI)		VE at 7 months after the last dose (95% CI)		*P* value of the vaccine sequence-time interaction		Months since the last dose to VE=50%
**2-dose vaccination**										
	**VE against severe COVID-19**									
		SV^b^ + AZ^c^	55.7 (53.6-57.7)		47.8 (45.7-49.8)		<.001		5.7	
		AZ + PZ^d^	54.8 (5.3-58.8)		43.6 (39.2-47.7)		.01		4.3	
	**VE against fatal COVID-19**									
		SV + AZ	47.4 (44.1-5.5)		45.8 (42.9-48.6)		.36		Omitted^e^	
		AZ + PZ	54.0 (48.0-59.3)		5.9 (45.9-55.4)		.58		Omitted^e^	
**3-dose vaccination**										
	**VE against severe COVID-19**									
		SV-SV-AZ	81.2 (78.6-83.5)		82.3 (8.3-84.0)		.55		Omitted^f^	
		SV-AZ-AZ	76.2 (72.3-79.5)		66.0 (61.9-69.6)		.11		Omitted^f^	
		SV-AZ-PZ	76.0 (73.3-78.4)		58.5 (55.0-61.8)		<.001		8.7	
		SP^g^-SP-PZ	79.8 (76.2-82.9)		68.2 (63.9-71.9)		.02		11.9	
		AZ-AZ-PZ	86.5 (85.3-87.7)		71.6 (69.6-73.6)		<.001		1.8	
		AZ-AZ-MN^h^	88.8 (86.5-9.8)		72.4 (68.3-75.9)		<.001		1.3	
	**VE against fatal COVID-19**									
		SV-SV-AZ	86.2 (82.8-89.0)		86.3 (83.6-88.6)		.98		Omitted^f^	
		SV-AZ-AZ	72.0 (66.3-76.6)		61.1 (55.4-66.2)		.22		Omitted^f^	
		SV-AZ-PZ	77.9 (74.6-8.8)		6.3 (55.8-64.4)		.002		9.0	
		SP-SP-PZ	76.5 (7.8-81.1)		73.2 (68.5-77.3)		.64		Omitted^f^	
		AZ-AZ-PZ	88.8 (87.3-9.1)		79.7 (77.7-81.6)		<.001		14.6	
		AZ-AZ-MN	92.4 (89.8-94.3)		87.3 (84.2-89.7)		.18		Omitted^e^	

^a^VE: vaccine effectiveness at a particular month estimated by the vaccine sequence-time interaction formula using the β coefficients adjusted for sex, age group, living province, and month of vaccination.

^b^SV: Sinovac (CoronaVac).

^c^AZ: AstraZeneca (ChAdOx1).

^d^PZ: Pfizer/BioNTech (BNT162b2).

^e^T_VE=50%_ was omitted because the initial vaccine effectiveness was not significantly greater than 50% protective effectiveness.

^f^T_VE=50%_ was omitted due to no evidence of vaccine effectiveness waning within 1 year.

^g^SP: Sinopharm (BBIBP-CorV).

^h^MN: Moderna (mRNA-1273).

### Subgroup Analysis

Figures S3-S18 of Multimedia Appendix 1 display the decline in VE over time, stratified by age group, and Table 2 provides a comprehensive summary of these figures. This table presents the effectiveness of the 2- and 3-dose COVID-19 vaccine sequences stratified by age group in relation to severe COVID-19 cases and COVID-19-related fatalities. The initial VE was compared with the VE 7 months after administration of the last dose. These findings indicate that VE is generally lower in people aged >80 years. The VE of distinct vaccine sequences varied based on the age group and outcome; however, the *P* values suggest that these differences were not statistically significant. The SV-SV-AZ regimen provided at least 6 months of durable VE against both severe and fatal COVID-19 in all age groups, including in those aged over 80 years (Figures S7-S8 of Multimedia Appendix 1). Neither of the dose regimens included in our analysis (SV-AZ and AZ-PZ) provided protective efficacy (>50% VE). Conversely, the VE of each 3-dose sequence increased (>50%) and subsequently waned over time, with a significant *P* value for the interaction term. Those older (age 61-80 years and >80 years) experienced reduction in VE to below 50% against severe or fatal COVID-19 within 7 months of the last dose, except for the SV-SV-AZ sequence.

## Discussion

### Principal Findings

In Thailand, as in many other LMICs, most people receive heterologous vaccine regimens for primary series and booster doses. The main 2-dose regimens delivered in Thailand (SV-AZ and AZ-PZ) exhibited suboptimal protection against severe COVID-19, with VE decreasing to <50% after 5-7 months, as confirmed by the Mantel-Haenszel method and logistic regression. Furthermore, the initial effectiveness of these vaccines against COVID-19-related mortality was <50%. In contrast, the 3-dose regimen provided superior initial protection against both severe and fatal COVID-19, with the VE remaining >50% for at least 8 months, as determined by logistic regression with durability time-interaction modeling. However, among individuals aged ≥80 years, all vaccine combinations conferred a comparatively lower level of protection with shorter durability relative to other age groups. For the 2-dose regimen in Thailand, sequential administration of the SV-AZ vaccine was initially favored even after the availability of AZ-AZ because of a shorter recommended interdose interval based on research examining humoral immune responses [5,38]. Nevertheless, a subsequent test-negative case-control study suggested that SV-AZ was ineffective against severe COVID-19 (VE<50%) within 3 months of administering the final dose [17], which is consistent with our findings. Regarding the 3-dose regimens, other studies from Thailand similarly found heterologous vaccines to have a high VE, although they included a different set of regimens in their analysis [18].

In light of a heterologous 3-dose vaccination strategy that provides a minimum of 8 months of sustained VE against severe and fatal COVID-19 cases, the need for annual immunization has become a topic of ongoing debate [39,40], particularly given the financial burden of regular COVID-19 vaccination campaigns. Information from this study can potentially help governments identify appropriate target groups and vaccination frequencies for future COVID-19 booster vaccination policies. The durability of immunity acquired through vaccination is known to be nonuniform across different age groups due to immunosenescence [41]. Consequently, older individuals may not develop the same level of immunity against COVID-19 as younger adults, rendering vaccines less effective in preventing severe and fatal COVID-19 [42-45]. Our data show that VE for individuals aged >80 years waned after 6 months. Only the SV-SV-AZ regimen exceptionally provided more than 50% effectiveness against both severe and fatal COVID-19 over 6 months. Therefore, individuals aged >80 years receiving other regimens might need a booster dose approximately every 6 months during the pandemic era.

### Limitations

This study has a few limitations that are primarily related to the use of national registry data. We were unable to identify information on unmeasured confounders such as occupations, comorbidities, and SARS-CoV-2 variants. In Thailand, health care workers were prioritized at the start of the campaign, and as it would be expected that health care workers might have different risks of developing severe COVID-19, this may have led to overestimates or underestimates of VE of the SV-AZ regimen in our study [46]. Similarly, we could not control for the comorbidities that increase the risk of developing severe COVID-19, such as diabetes and immunocompromising conditions. Individuals with these comorbidities had priority for receiving vaccination [46]. Lack of control for comorbidities may have led to an underestimation of the VE of all vaccines.

Additionally, we were unable to control for the variants of concern, which is important because there was a switch from Delta to Omicron during the study period. However, partitioning the analysis into windows for the months when Alpha and Delta variants were dominant was not possible owing to an inadequate number of cases in the vaccinated population. Most of the Thai population only received their first dose of vaccine after the Delta variant surged. Booster doses were administered mainly in 2022 during the Omicron variant–dominant period. Therefore, in this study, the evaluation of VE applies mainly to protection against the Delta variant.

Another limitation of the registry data is that it was not possible to verify whether all the individuals in the cohort were truly uninfected. Therefore, it is likely that there were many individuals with hybrid immunity and undetected natural infections in the cohort. Furthermore, this study did not account for reinfection, which might lead to a survival bias, particularly in older populations, as the remaining population is likely to be healthier [47,48]. Finally, we were unable to continue our analysis beyond July 2022 when the CO-WARD registry database ended. We also did not track individuals after any evidence of SARS-CoV-2 infection; thus, we do not have data on reinfection. The lack of reinfection data means that this study does not provide information on the protective effect of natural SARS-CoV-2 infection against subsequent infections. Given the transmissibility of the Omicron variant, which frequently causes breakthrough infections in vaccinated individuals but is less virulent [49], it is possible that by 2024, natural immunity acquired from natural infection with the Omicron variant will protect most people from reinfection. Therefore, another study is needed to clarify whether an additional booster dose is needed for individuals younger than 80 years.

### Conclusion

Our findings show evidence of a significant waning of vaccine protection in Thailand, particularly for the main heterologous 2-dose regimens (SV-AZ and AZ-PZ). Overall, all 3-dose regimens demonstrated higher VE than 2-dose regimens and maintained durability of VE against severe and fatal COVID-19, with over 50% effectiveness for at least 8 months. Only the SV-SV-AZ regimen consistently showed effectiveness across all age groups. Individuals older than 80 years who received other regimens should receive a booster dose at 6 months after their last dose. Within a 7-month follow-up period, the heterologous prime-boost vaccination strategies, which involve a primary series of an inactivated virus vaccine and a booster with either a viral vector or mRNA vaccine, is proven to prevent severe and fatal COVID-19 in situations of vaccine shortages. This approach is particularly pertinent in scenarios, wherein viral vector and mRNA vaccines are not readily available during the early stages of a pandemic. However, estimates of VE over a year are needed for vaccine regimens delivered in Thailand to inform future vaccination policies. The ongoing emergence of SARS-CoV-2 variants and the resulting herd immunity warrant the continuation of vaccine development and monitoring of VE.

## Appendix

Multimedia Appendix 1 Supplementary data.

## Abbreviations

aOR: adjusted odds ratio
ATK: antigen test kit
AZ: AstraZeneca (ChAdOx1 nCoV-19)
LMIC: low- and middle-income country
MN: Moderna (mRNA-1273)
MoPH: Ministry of Public Health
mRNA: messenger ribonucleic acid
PZ: Pfizer/BioNTech (BNT162b2)
RT-PCR: reverse transcription polymerase chain reaction
SP: Sinopharm (BBIBP-CorV)
SV: Sinovac (CoronaVac)
VE: vaccine effectiveness

## Data Availability

The aggregated data and R codes are shared by GitHub in [50]. To accommodate readers who are not familiar with R coding, we have also provided an Excel file that summarizes the results for each time period in [51].
