# Supplementary material for: Durability of the Effectiveness of Heterologous COVID-19 Vaccine Regimens in Thailand: Retrospective Cohort Study Using National Registration Data
Source: JMIR Public Health Surveill. 2024 Mar 5;10:e48255. doi: 10.2196/48255 (PMC10951833; doi:10.2196/48255)
Supplement: Multimedia Appendix 1 [file publichealth_v10i1e48255_app1.docx]

# **Supplementary data**

# Figures


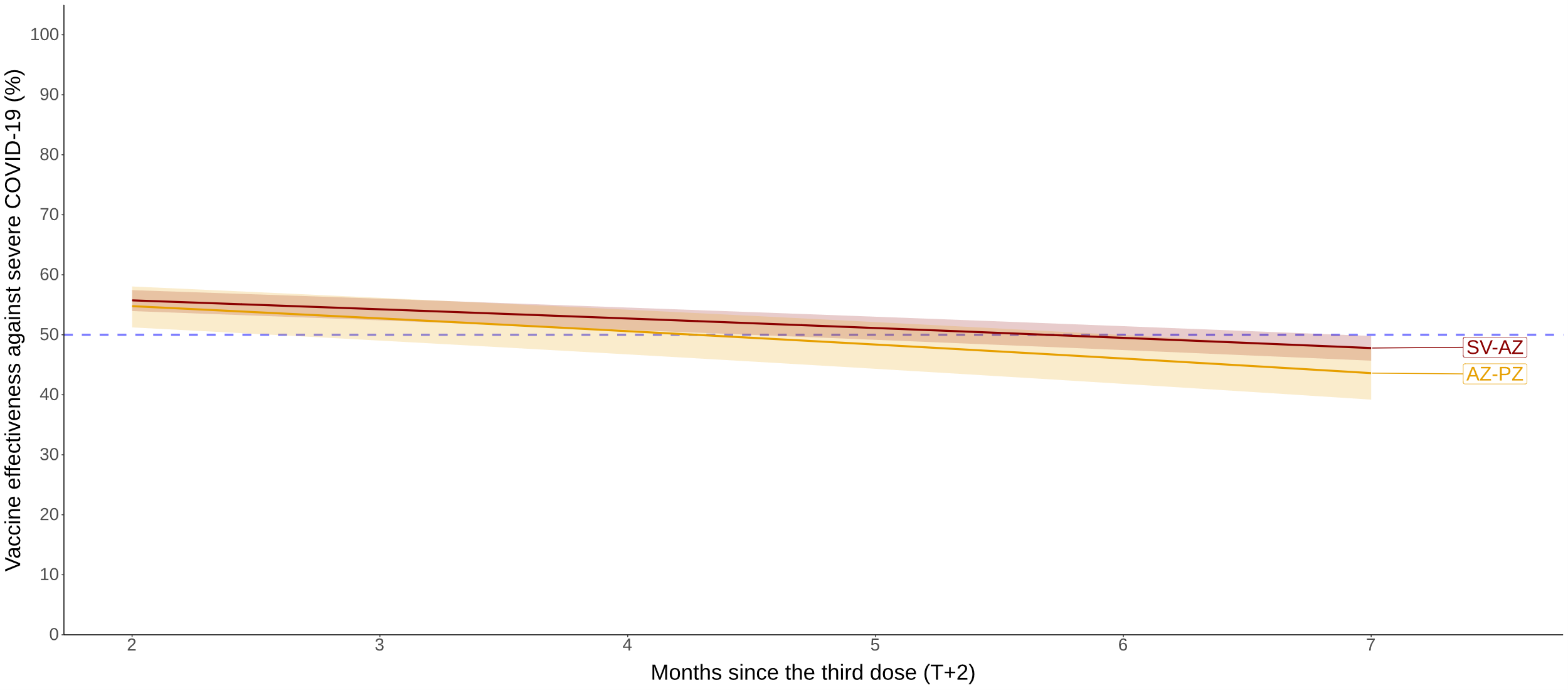


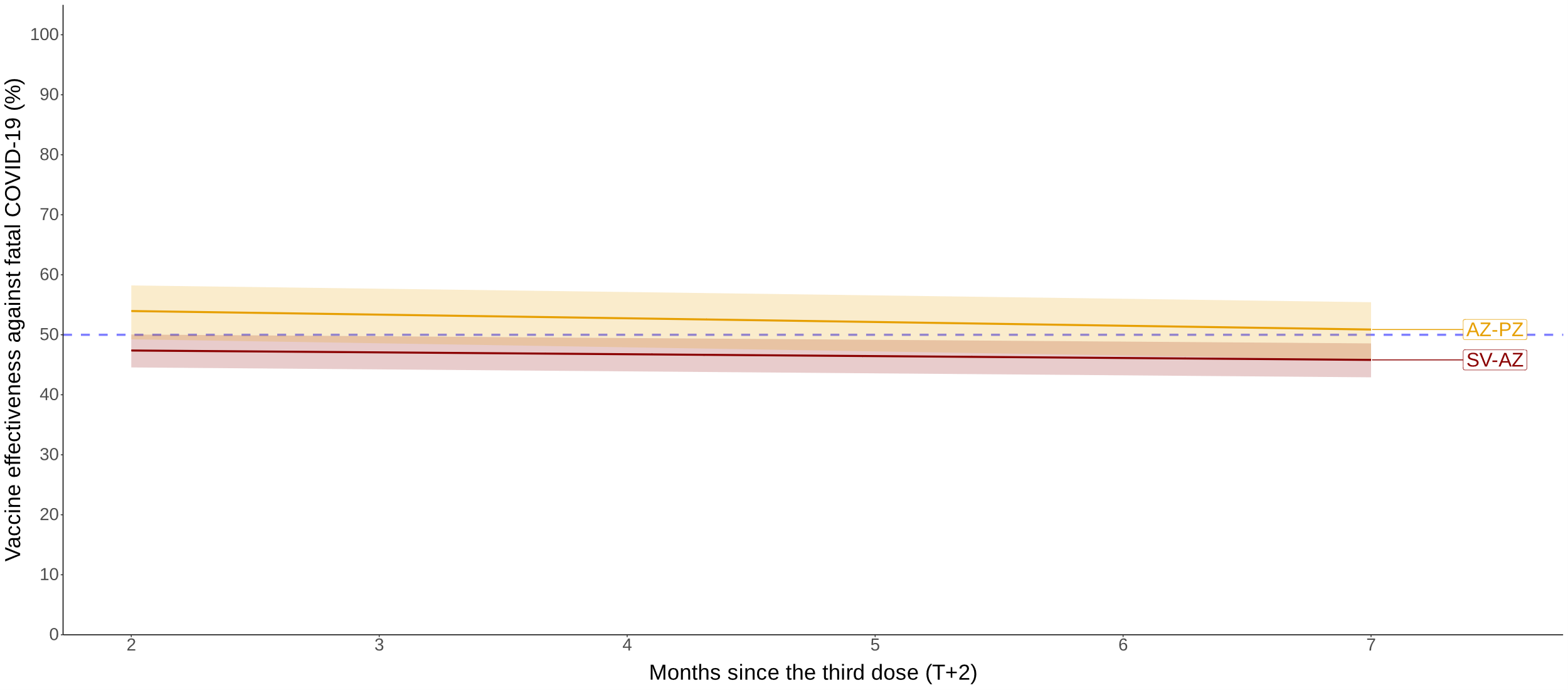


**Figure S1.** Vaccine effectiveness of 2-dose vaccine sequences against severe and fatal COVID-19 was estimated by the logistic regression method from July 2021 to July 2022.
AZ, AstraZeneca (ChAdOx1); PZ, Pfizer/BioNTech (BNT162b2); SV, Sinovac (CoronaVac)


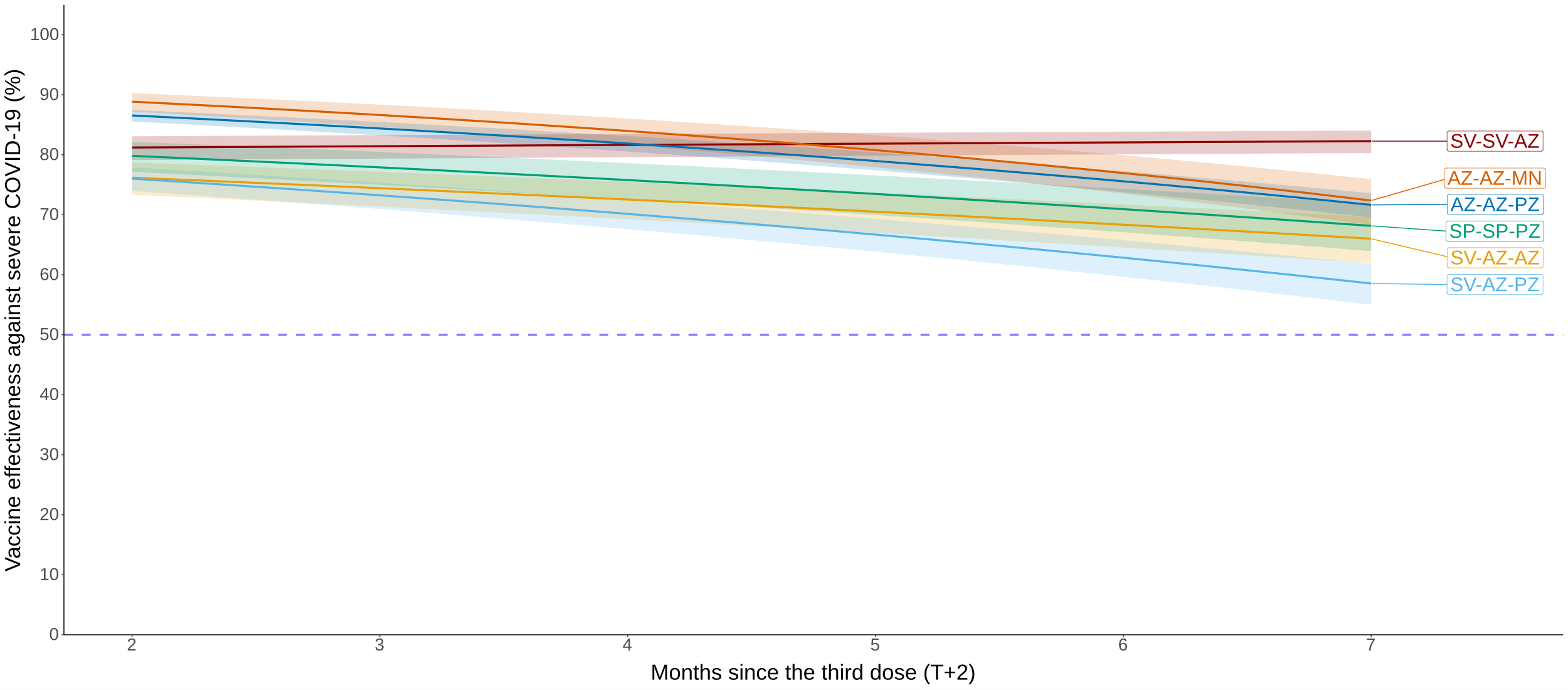

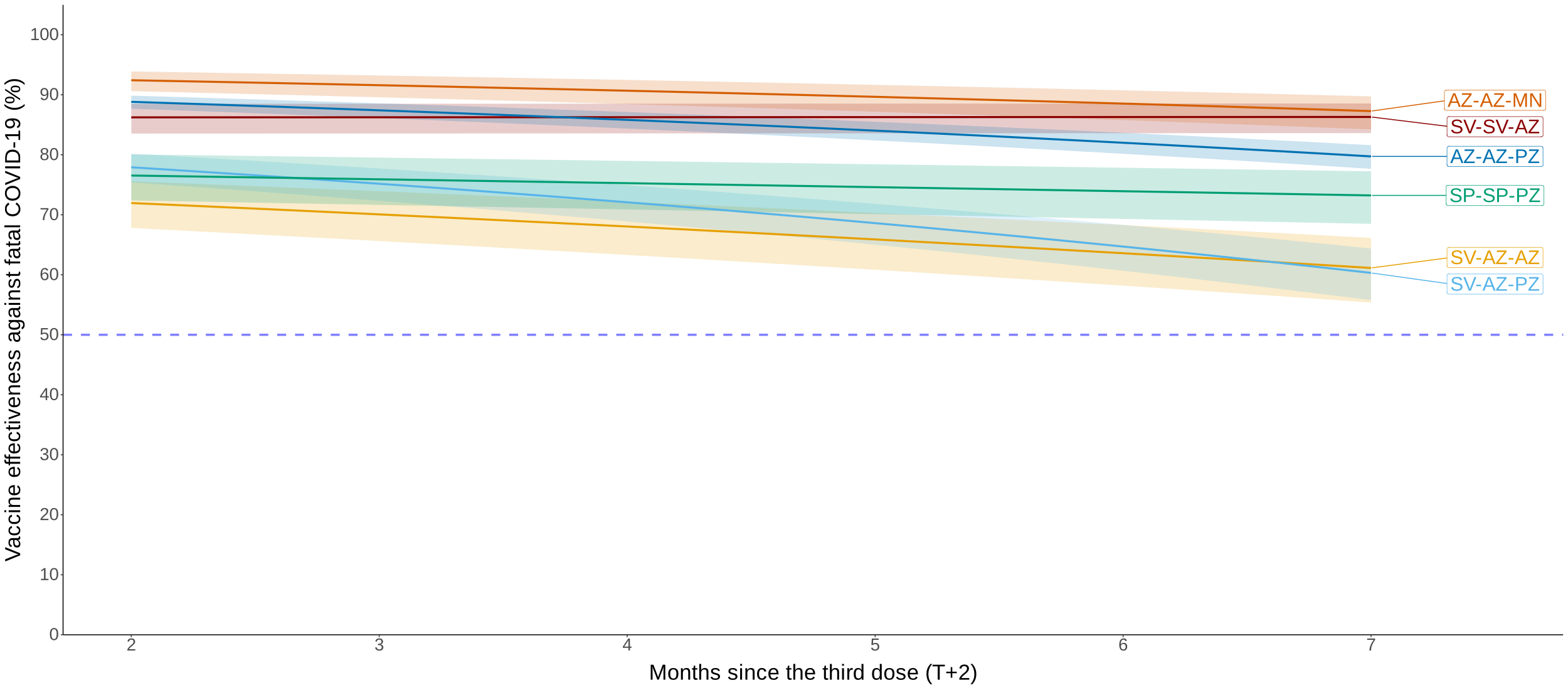


**Figure S2.** Vaccine effectiveness of 3-dose vaccine sequences against severe and fatal COVID-19 was estimated using the Mantel–Haenszel risk ratio method from July 2021 to July 2022.
AZ, Astrazeneca (ChAdOx1); MN, Moderna (mRNA-1273); PZ, Pfizer/BioNTech (BNT162b2); SP, Sinopharm (BBIBP-CorV); SV, Sinovac (CoronaVac)


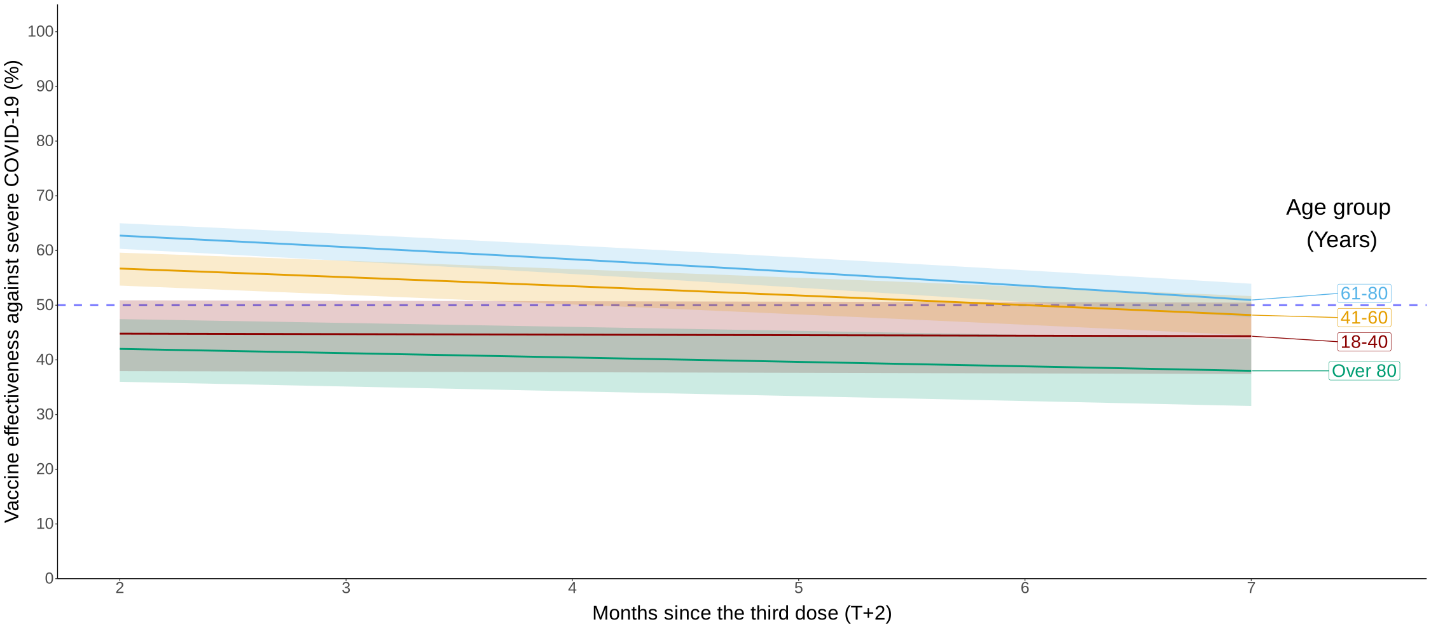


**Figure S3.** Effectiveness of the SV-AZ sequence against severe COVID-19 stratified by age group from July 2021 to July 2022. AZ, AstraZeneca (ChAdOx1); SV, Sinovac (CoronaVac)

**
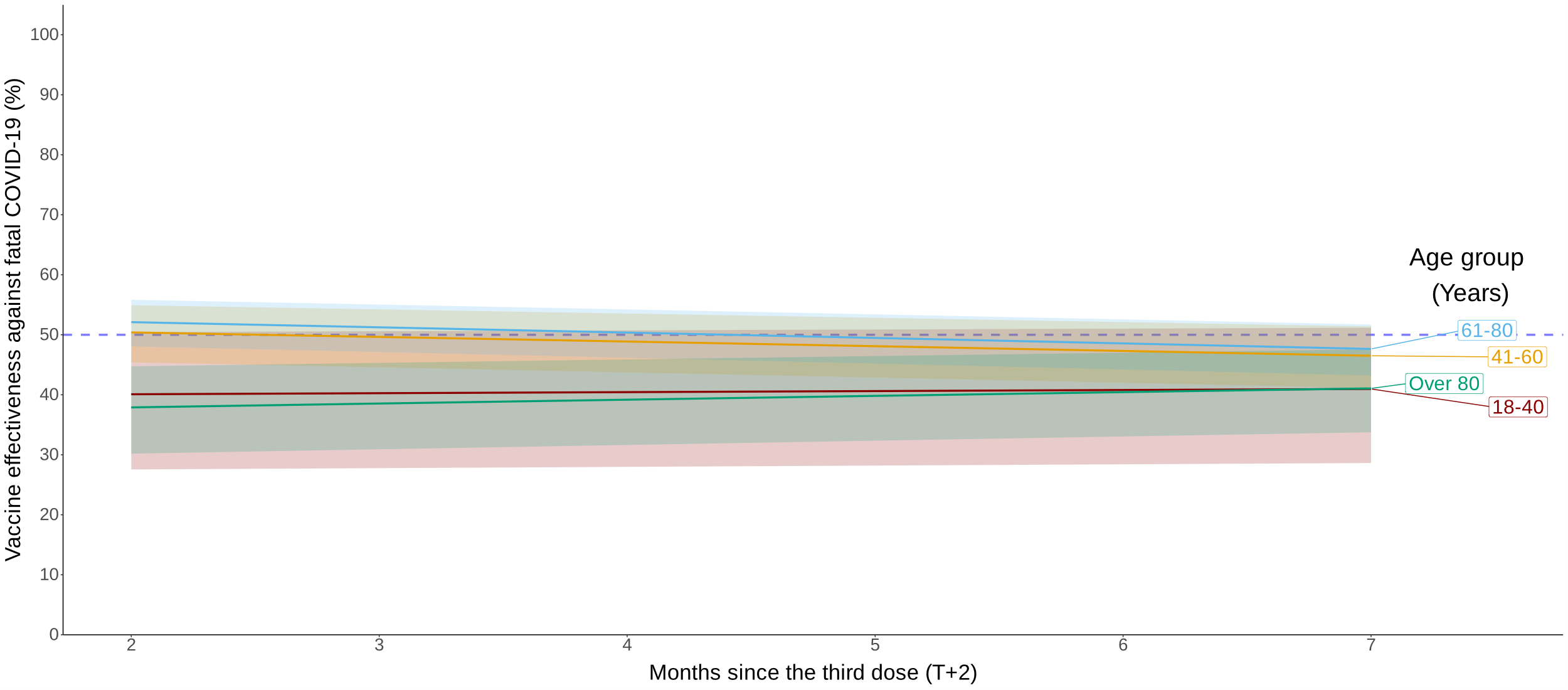
**

**Figure S4.** Effectiveness of the SV-AZ sequence against fatal COVID-19 stratified by age group from July 2021 to July 2022. AZ, AstraZeneca (ChAdOx1); SV, Sinovac (CoronaVac)


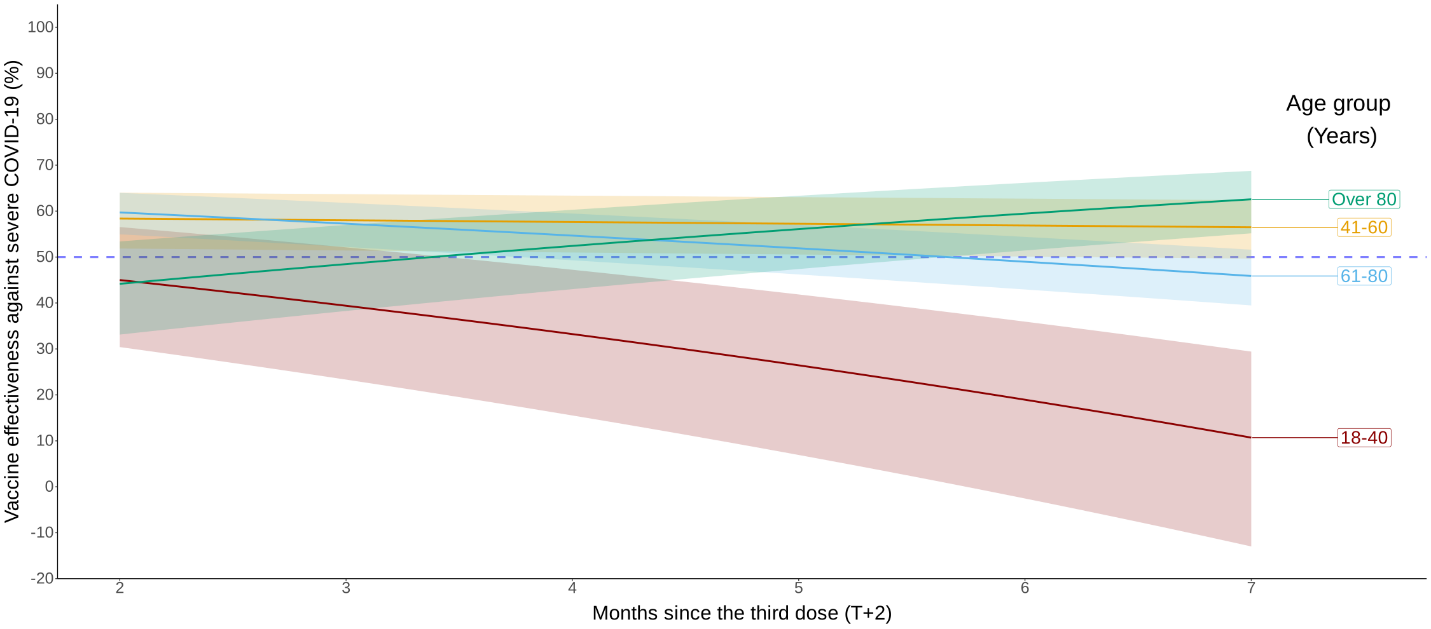
**Figure S5.** Effectiveness of the AZ-PZ sequence against severe COVID-19 stratified by age group from July 2021 to July 2022. AZ, AstraZeneca (ChAdOx1); PZ, Pfizer/BioNTech (BNT162b2)

**
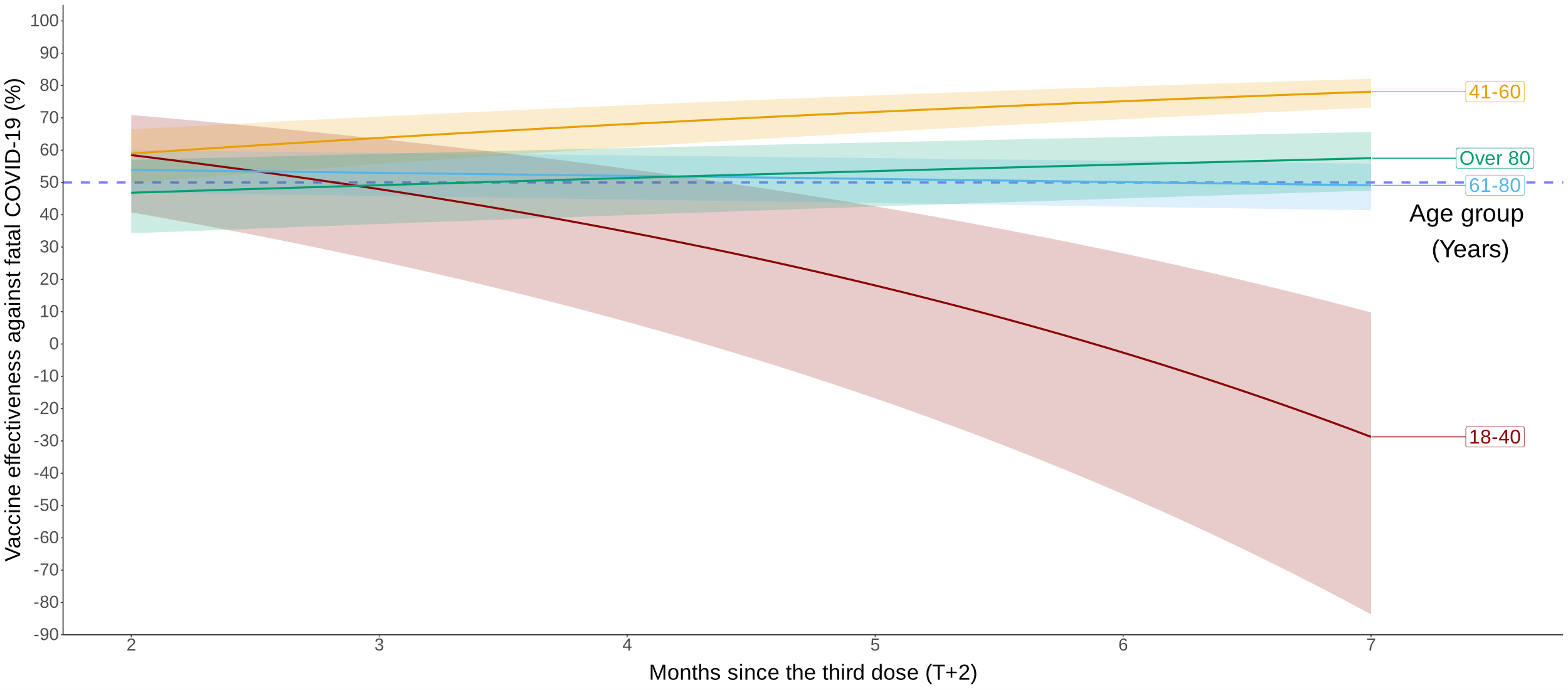
**

**Figure S6**. Effectiveness of the AZ-PZ sequence against fatal COVID-19 stratified by age group from July 2021 to July 2022. AZ, AstraZeneca (ChAdOx1); PZ, Pfizer/BioNTech (BNT162b2)


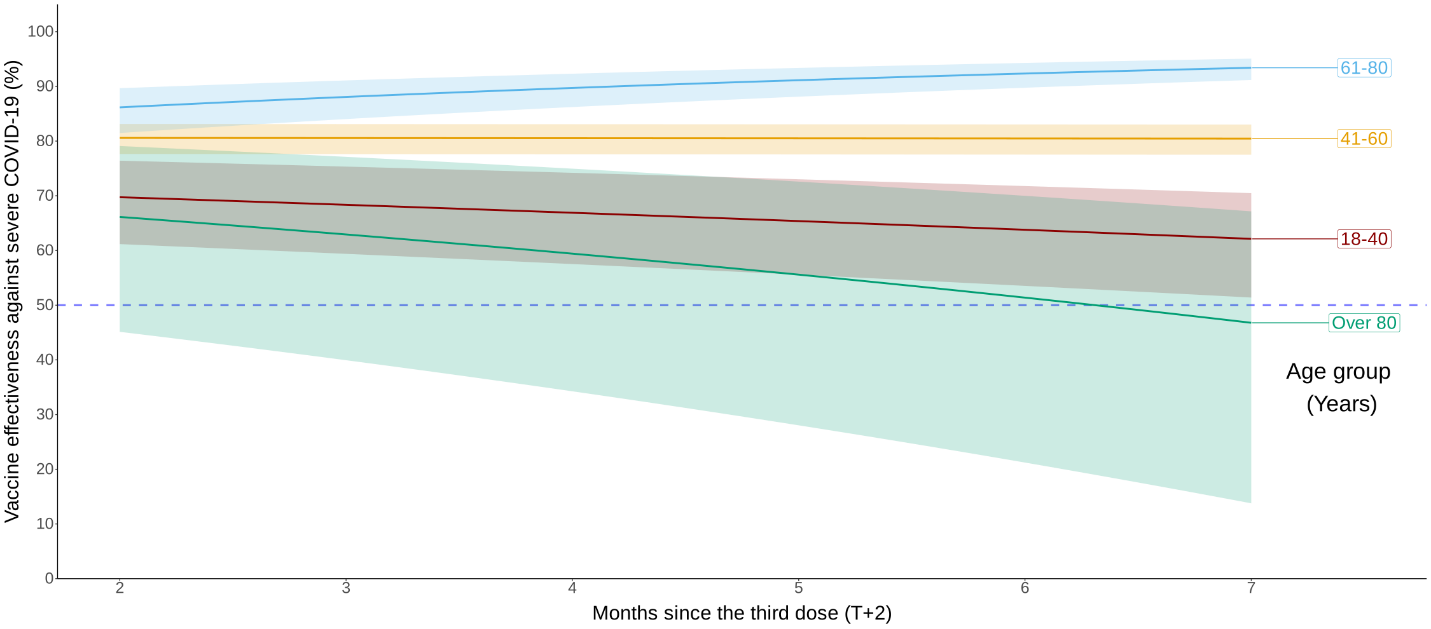
**Figure S7.** Effectiveness of the SV-SV-AZ sequence against severe COVID-19 stratified by age group from July 2021 to July 2022. AZ, AstraZeneca (ChAdOx1); SV, Sinovac (CoronaVac)


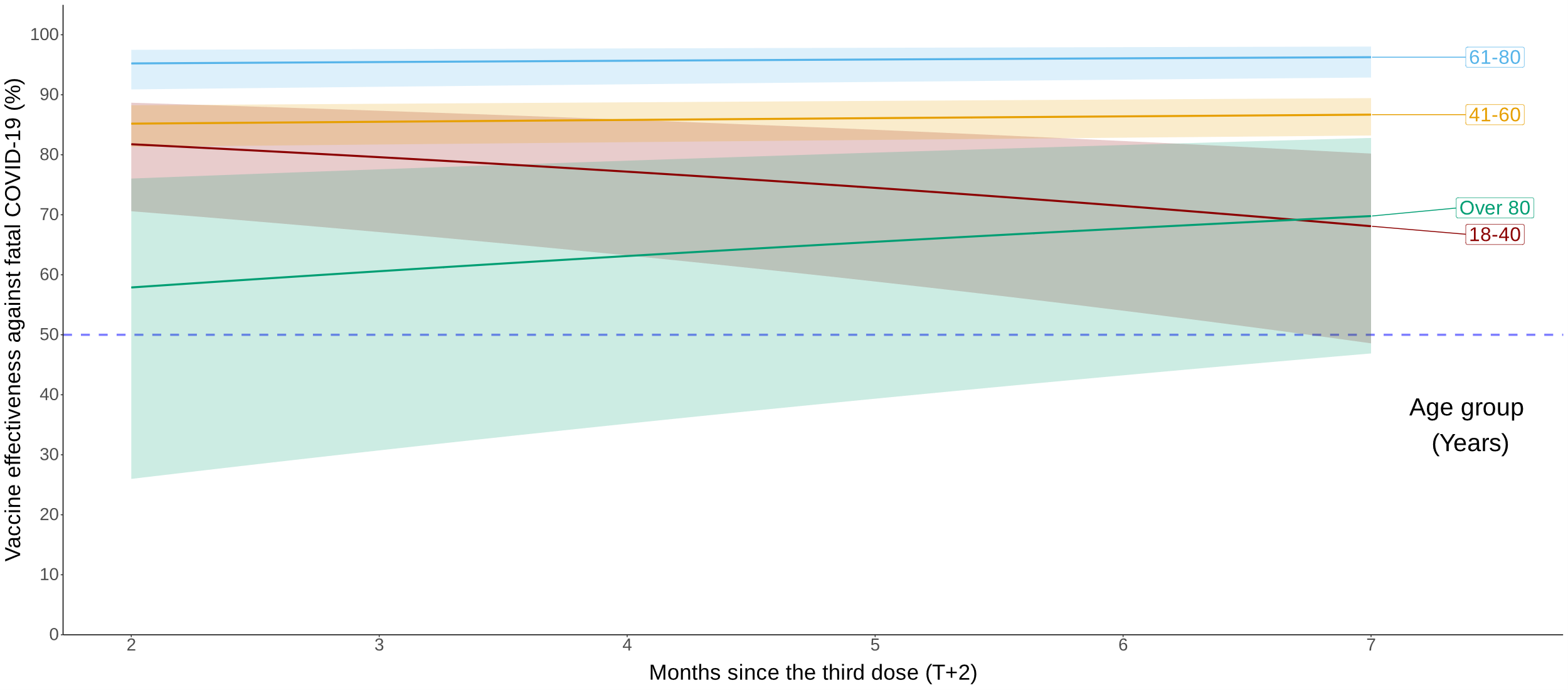
**Figure S8.** Vaccine effectiveness of the SV-SV-AZ sequence against fatal COVID-19 stratified by age group from July 2021 to July 2022. AZ, AstraZeneca (ChAdOx1); SV, Sinovac (CoronaVac)


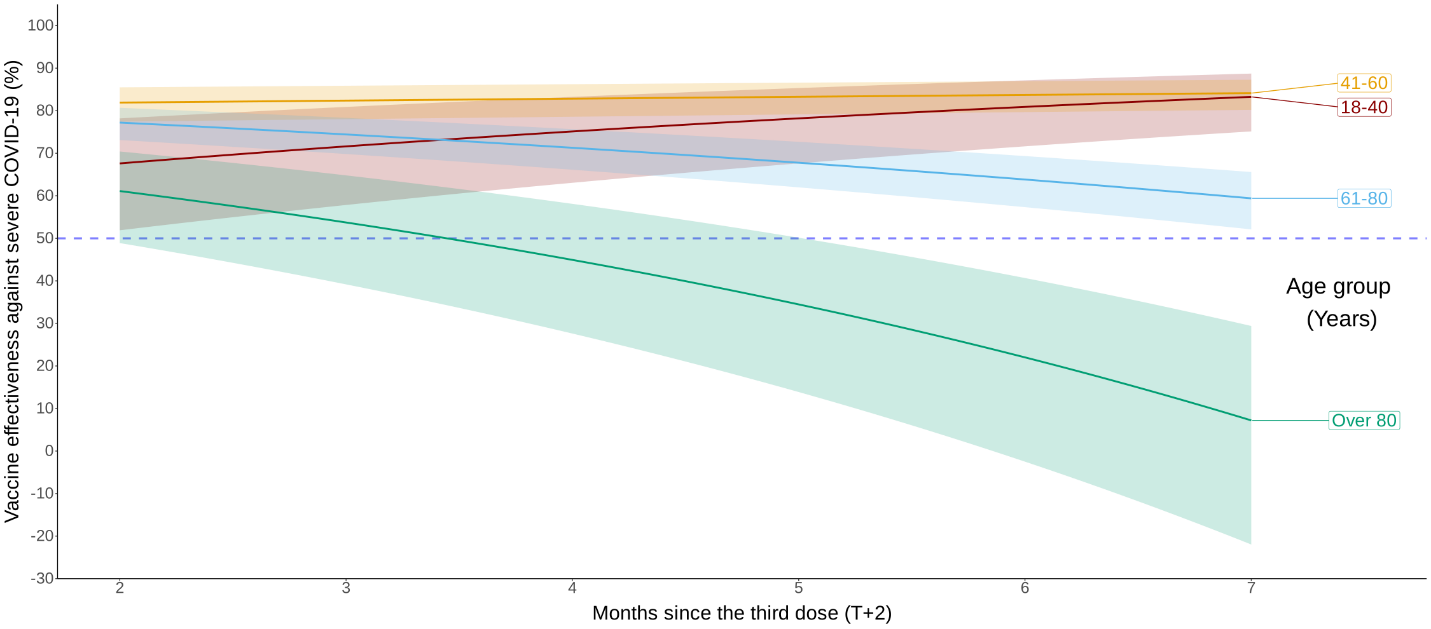


**Figure S9.** Effectiveness of the SV-AZ-AZ sequence against severe COVID-19 stratified by age group from July 2021 to July 2022. AZ, AstraZeneca (ChAdOx1); SV, Sinovac (CoronaVac)

**
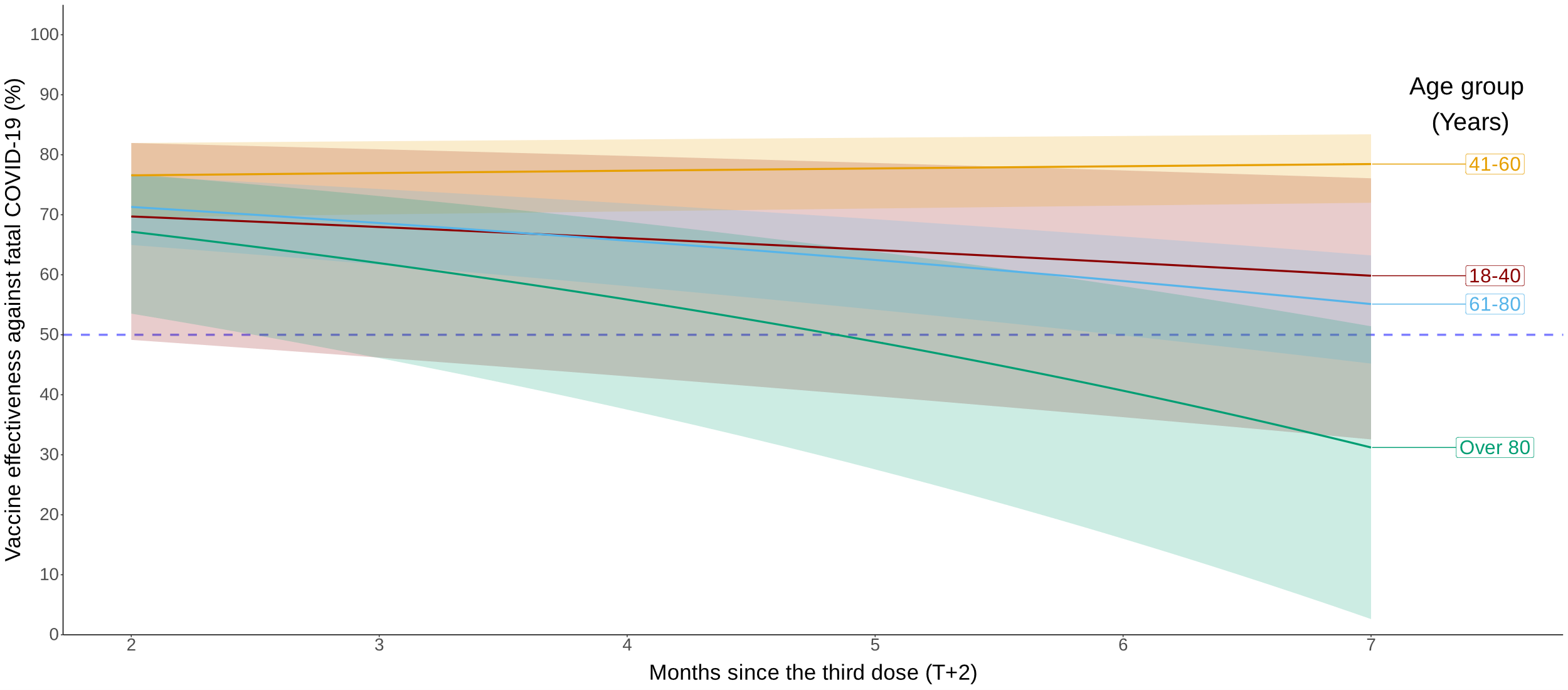
**

**Figure S10.** Effectiveness of the SV-AZ-AZ sequence against fatal COVID-19 stratified by age group from July 2021 to July 2022. AZ, AstraZeneca (ChAdOx1); SV, Sinovac (CoronaVac)


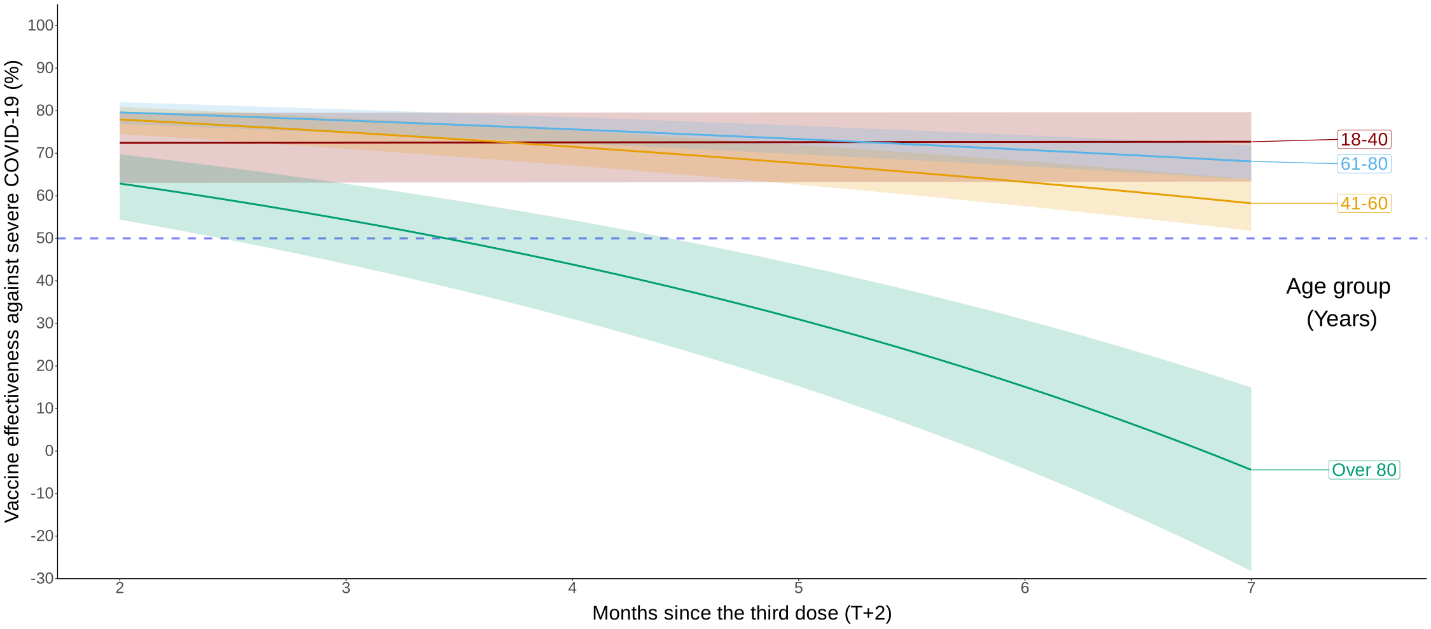


**Figure S11.** Effectiveness of the SV-AZ-PZ sequence against severe COVID-19 stratified by age group from July 2021 to July 2022. AZ, AstraZeneca (ChAdOx1); PZ, Pfizer/BioNTech (BNT162b2); SV, Sinovac (CoronaVac)

**
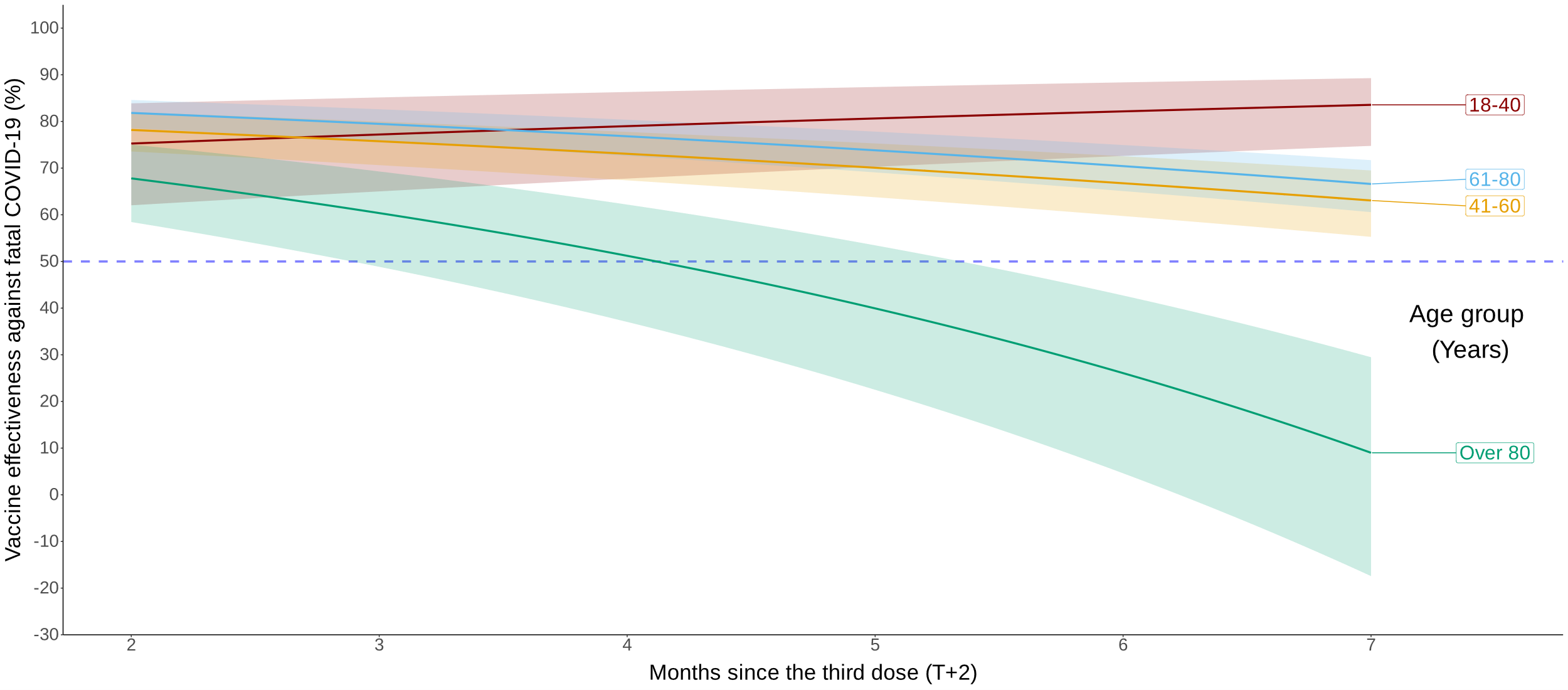
**

**Figure S12.** Vaccine effectiveness of the SV-AZ-PZ sequence against fatal COVID-19 stratified by age group from July 2021 to July 2022. AZ, AstraZeneca (ChAdOx1); PZ, Pfizer/BioNTech (BNT162b2); SV, Sinovac (CoronaVac)


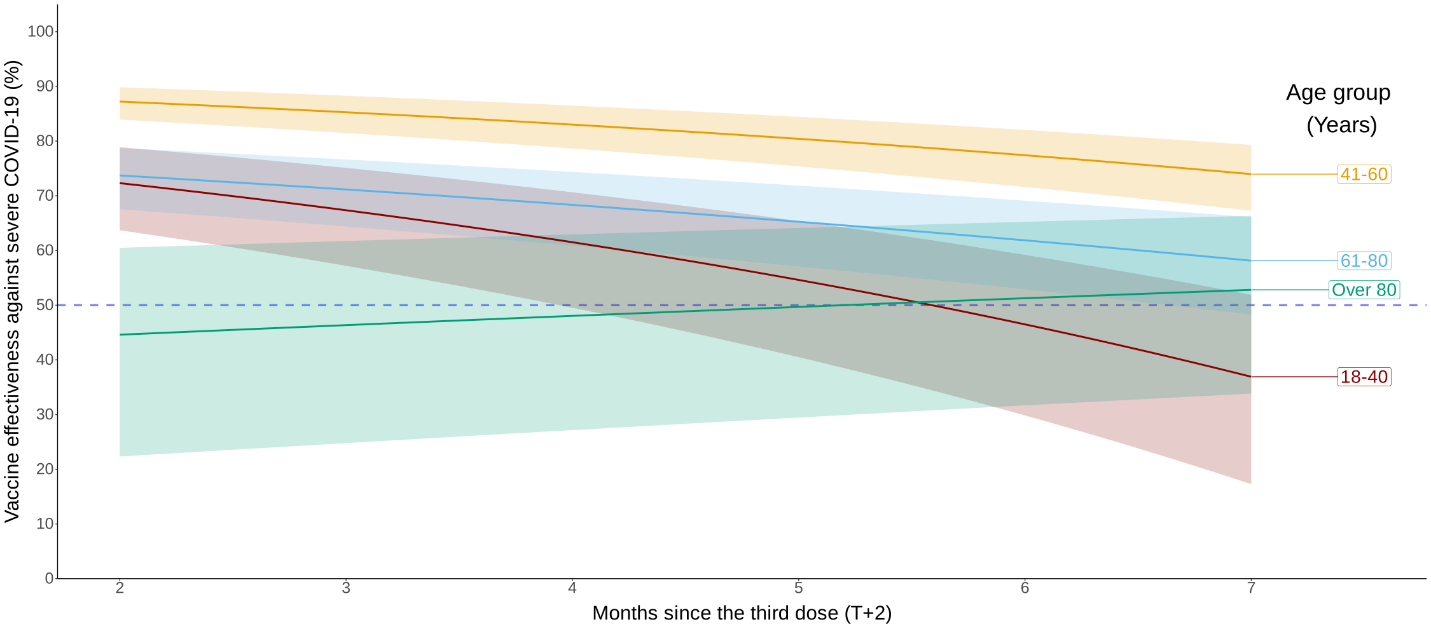


**Figure S13.** Effectiveness of the SP-SP-PZ sequence against severe COVID-19 stratified by age group from July 2021 to July 2022. PZ, Pfizer/BioNTech (BNT162b2); SP, Sinopharm (BBIBP-CorV)

**
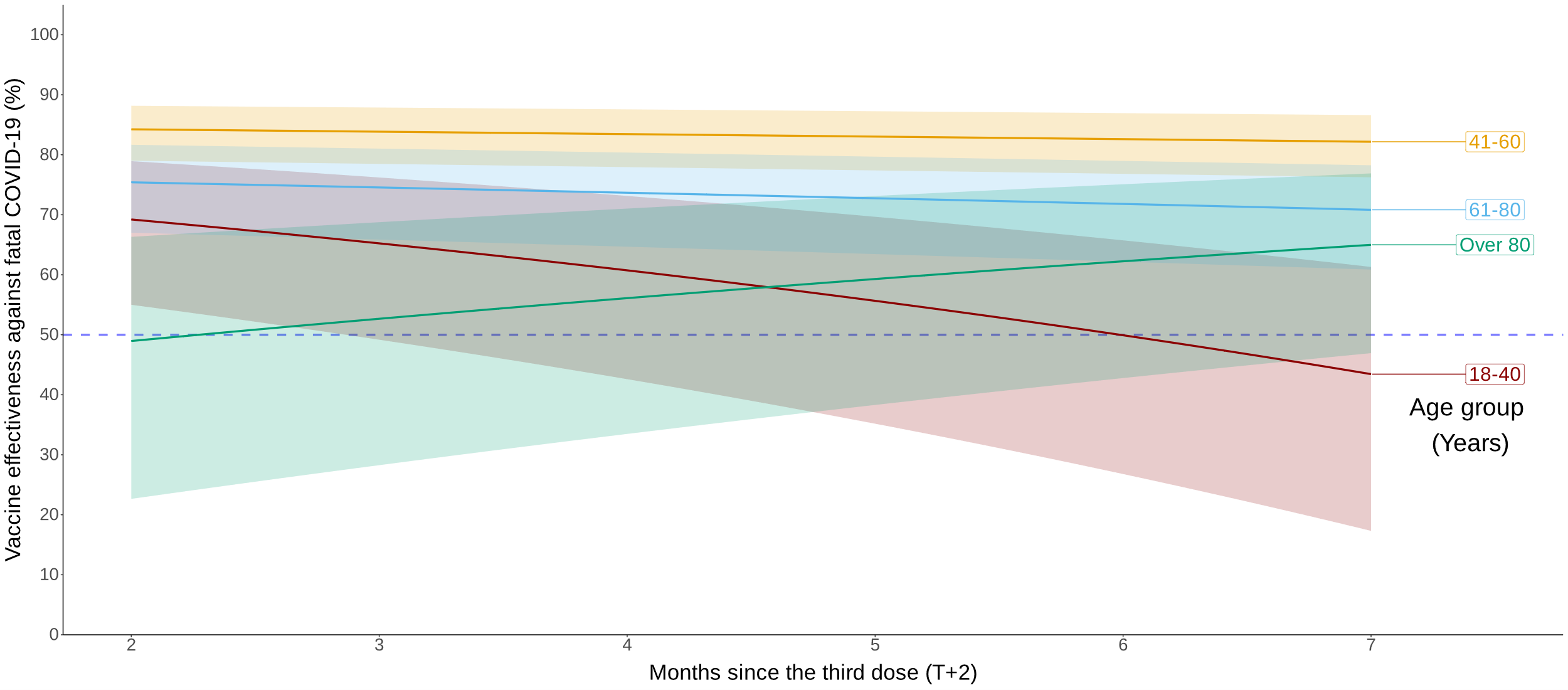
**

**Figure S14.** Effectiveness of the SP-SP-PZ sequence against fatal COVID-19 stratified by age group from July 2021 to July 2022. PZ, Pfizer/BioNTech (BNT162b2); SP, Sinopharm (BBIBP-CorV)

**
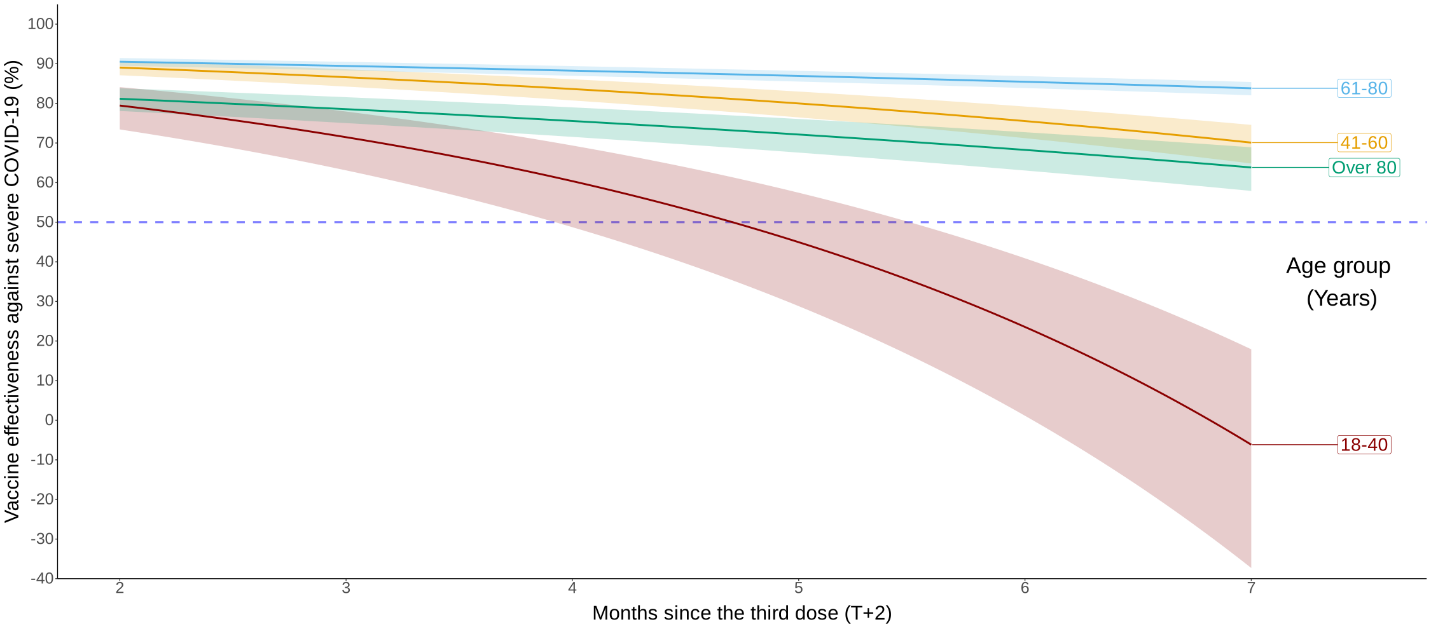
**

**Figure S15.** Effectiveness of the AZ-AZ-PZ sequence against severe COVID-19 stratified by age group from July 2021 to July 2022. AZ, AstraZeneca (ChAdOx1); PZ, Pfizer/BioNTech (BNT162b2)


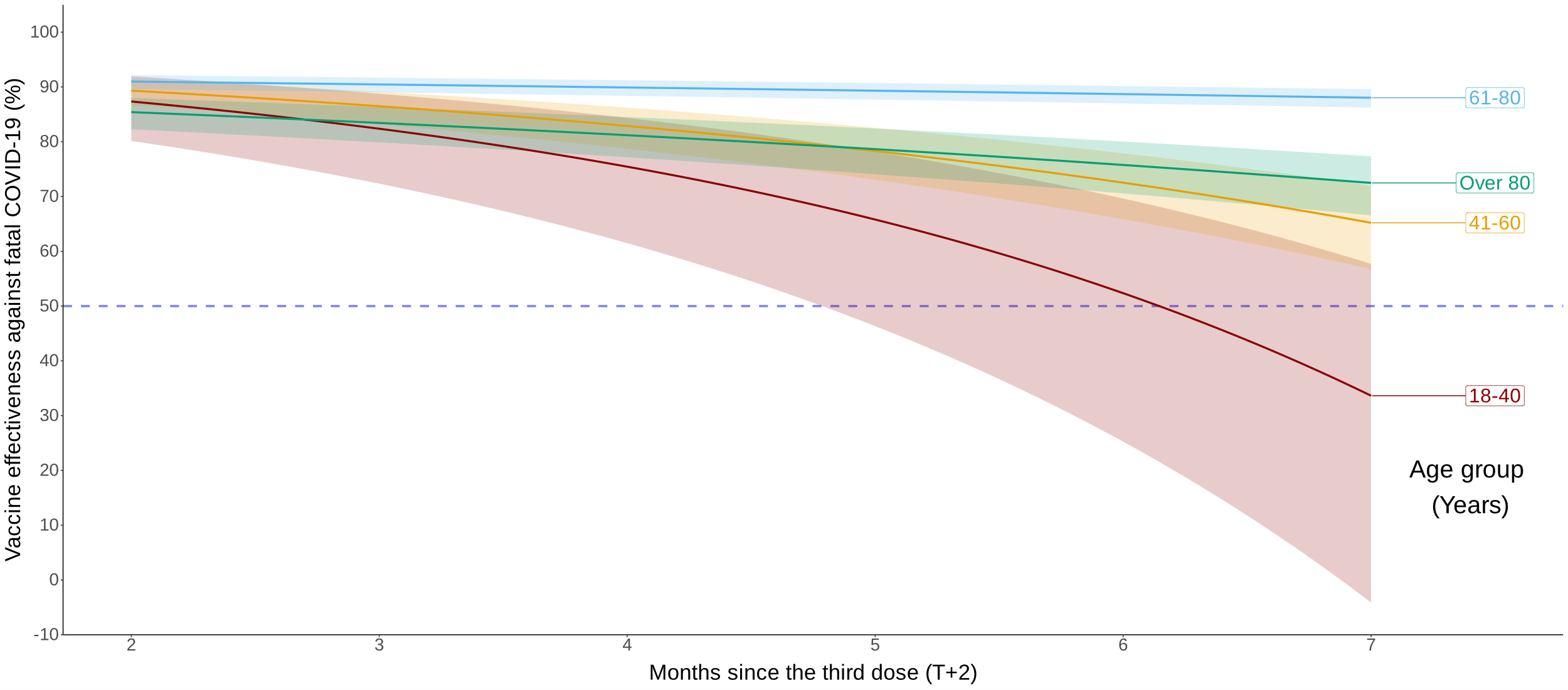
**Figure S16.** Effectiveness of the AZ-AZ-PZ sequence against fatal COVID-19 stratified by age group from July 2021 to July 2022. AZ, AstraZeneca (ChAdOx1); PZ, Pfizer/BioNTech (BNT162b2)


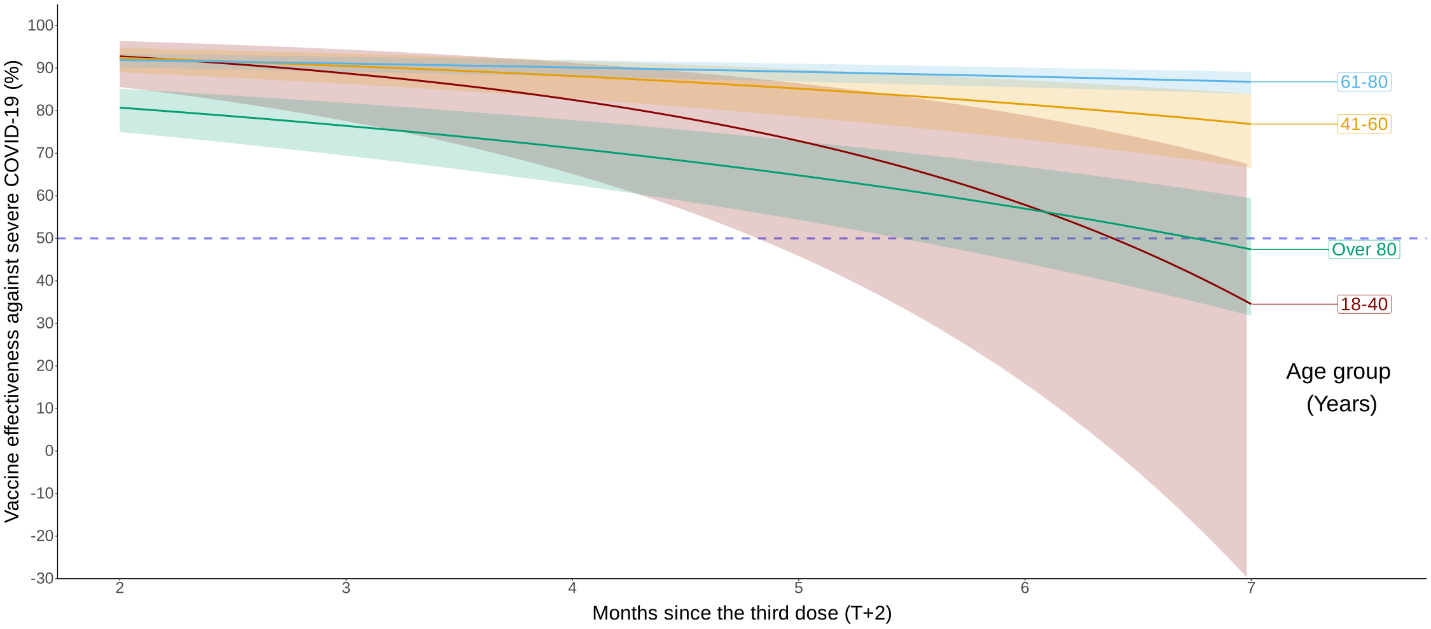


**Figure S17.** Effectiveness of the AZ-AZ-MN sequence against severe COVID-19 stratified by age group from July 2021 to July 2022. AZ, Astrazeneca (ChAdOx1), MN, Moderna (mRNA-1273)


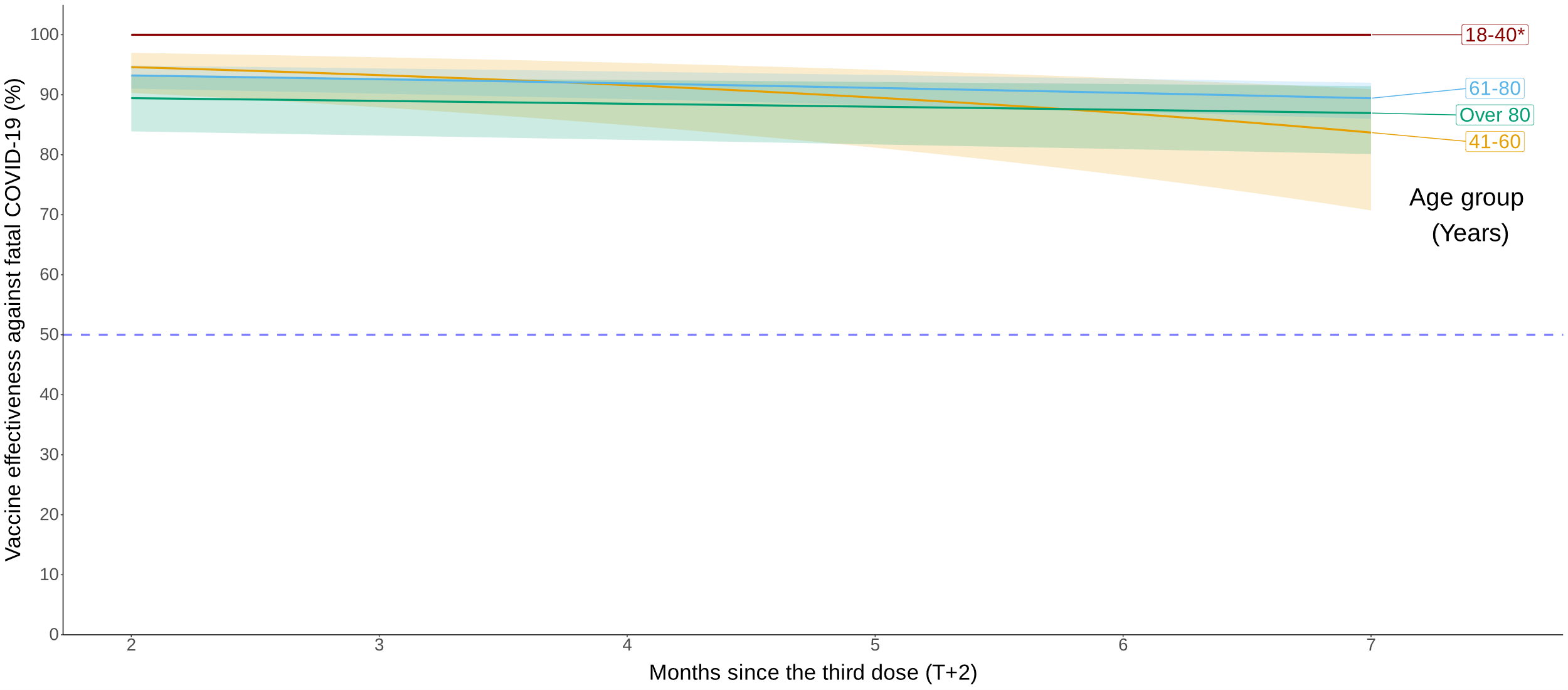
**Figure S18.** Vaccine effectiveness of the AZ-AZ-MN sequence against fatal COVID-19 stratified by age group from July 2021 to July 2022. AZ, Astrazeneca (ChAdOx1), MN, Moderna (mRNA-1273)

# Tables

**Table S1.** Variance inflation factors for each model.

| **Vaccine effectiveness** | **Vaccine sequence** | **Sex** | **Age group** | **Province of residence** | **Month of infection**^a^ |
| --- | --- | --- | --- | --- | --- |
| **2-dose vaccination** | |  |  |  |  |
| *against severe COVID-19* | |  |  |  |  |
|  | SV-AZ | 1.00 | 1.04 | 1.09 | 4.61 |
|  | AZ-PZ | 1.00 | 1.03 | 1.07 | 4.60 |
| *against fatal COVID-19* | |  |  |  |  |
|  | SV-AZ | 1.00 | 1.04 | 1.10 | 3.69 |
|  | AZ-PZ | 1.01 | 1.04 | 1.08 | 3.75 |
| **3-dose vaccination** |  |  |  |  |  |
| *against severe COVID-19* | |  |  |  |  |
|  | SV-SV-AZ | 1.00 | 1.04 | 1.14 | 4.56 |
|  | SV-AZ-AZ | 1.00 | 1.03 | 1.07 | 4.59 |
|  | SV-AZ-PZ | 1.00 | 1.03 | 1.07 | 4.67 |
|  | SP-SP-PZ | 1.00 | 1.04 | 1.06 | 4.57 |
|  | AZ-AZ-PZ | 1.00 | 1.04 | 1.09 | 4.76 |
|  | AZ-AZ-MN | 1.00 | 1.03 | 1.07 | 4.58 |
| *against fatal COVID-19* | |  |  |  |  |
|  | SV-SV-AZ | 1.01 | 1.04 | 1.10 | 3.72 |
|  | SV-AZ-AZ | 1.01 | 1.04 | 1.07 | 3.76 |
|  | SV-AZ-PZ | 1.00 | 1.04 | 1.07 | 3.80 |
|  | SP-SP-PZ | 1.01 | 1.04 | 1.07 | 3.74 |
|  | AZ-AZ-PZ | 1.01 | 1.04 | 1.10 | 3.83 |
|  | AZ-AZ-MN | 1.01 | 1.04 | 1.07 | 3.74 |

^a^Calendar month
AZ, Astrazeneca (ChAdOx1); MN, Moderna (mRNA-1273); PZ, Pfizer/BioNTech (BNT162b2); SP, Sinopharm (BBIBP-CorV); SV, Sinovac (CoronaVac)

**Table S2.** Vaccine effectiveness stratified by age group

| **Vaccine effectiveness** | **Vaccine sequence** | **Age group (years)** | **VE at 2 months after the last dose (95% CI)** | **VE at 7 months after the last dose**  **(95% CI)** | ***P*-value of the vaccine-time interaction** |  |
| --- | --- | --- | --- | --- | --- | --- |
| **2-dose vaccination** |  |  |  |  |  |  |
| *against severe COVID-19* |  |  |  |  |  |  |
|  | SV-AZ | 18–40 | 44.80 (36.64, 51.91) | 44.33 (37.43, 50.47) | 0.919 |  |
|  |  | 41–60 | 56.70 (53.04, 60.07) | 48.19 (44.47, 51.66) | <0.001 |  |
|  |  | 61–80 | 62.72 (59.90, 65.35) | 50.94 (47.78, 53.92) | <0.001 |  |
|  |  | >80 | 42.01 (34.81, 48.41) | 38.00 (31.57, 43.82) | 0.314 |  |
|  | AZ-PZ | 18–40 | 45.00 (25.90, 59.17) | 10.70 (-12.98, 29.42) | 0.102 |  |
|  |  | 41–60 | 58.38 (49.87, 65.44) | 56.48 (49.67, 62.37) | 0.835 |  |
|  |  | 61–80 | 59.71 (53.75, 64.91) | 45.88 (39.48, 51.60) | 0.010 |  |
|  |  | >80 | 44.17 (29.88, 55.55) | 62.57 (55.17, 68.75) | 0.094 |  |
| *against fatal COVID-19* |  |  |  |  |  |  |
|  | SV-AZ | 18–40 | 40.09 (25.20, 52.01) | 40.97 (28.63, 51.18) | 0.899 |  |
|  |  | 41–60 | 50.40 (44.55, 55.63) | 46.51 (41.13, 51.39) | 0.177 |  |
|  |  | 61–80 | 52.11 (47.36, 56.42) | 47.64 (43.22, 51.71) | 0.064 |  |
|  |  | >80 | 37.90 (28.65, 45.95) | 41.06 (33.75, 47.57) | 0.514 |  |
|  | AZ-PZ | 18–40 | 58.48 (35.11, 73.43) | -28.74 (-83.69, 9.77) | 0.003 |  |
|  |  | 41–60 | 58.95 (46.90, 68.27) | 78.08 (73.19, 82.08) | 0.064 |  |
|  |  | 61–80 | 53.90 (45.06, 61.31) | 49.12 (41.38, 55.84) | 0.517 |  |
|  |  | >80 | 46.82 (30.51, 59.30) | 57.52 (47.49, 65.63) | 0.404 |  |
| **3-dose vaccination** |  |  |  |  |  |  |
| *against severe COVID-19* |  |  |  |  |  |  |
|  | SV-SV-AZ | 18–40 | 69.74 (58.82, 77.77) | 62.13 (51.40, 70.48) | 0.295 | |
|  |  | 41–60 | 80.58 (76.94, 83.65) | 80.46 (77.51, 83.02) | 0.957 | |
|  |  | 61–80 | 86.17 (79.80, 90.53) | 93.40 (91.17, 95.07) | 0.040 | |
|  |  | >80 | 66.13 (37.75, 81.58) | 46.78 (13.78, 67.15) | 0.285 | |
|  | SV-AZ-AZ | 18–40 | 67.61 (46.77, 80.29) | 83.26 (75.14, 88.73) | 0.463 | |
|  |  | 41–60 | 81.91 (75.90, 86.42) | 84.13 (80.20, 87.27) | 0.782 | |
|  |  | 61–80 | 77.21 (71.64, 81.69) | 59.40 (52.11, 65.58) | 0.059 | |
|  |  | >80 | 61.11 (43.66, 73.16) | 7.20 (-21.96, 29.39) | 0.079 | |
|  | SV-AZ-PZ | 18–40 | 72.46 (59.84, 81.11) | 72.70 (63.35, 79.66) | 0.988 | |
|  |  | 41–60 | 77.96 (73.44, 81.67) | 58.24 (51.78, 63.83) | 0.011 | |
|  |  | 61–80 | 79.62 (76.10, 82.62) | 68.08 (63.85, 71.82) | 0.040 | |
|  |  | >80 | 62.88 (51.25, 71.74) | -4.43 (-28.20, 14.94) | 0.003 | |
|  | SP-SP-PZ | 18–40 | 72.31 (60.46, 80.61) | 36.91 (17.29, 51.88) | 0.038 | |
|  |  | 41–60 | 87.23 (82.61, 90.61) | 73.96 (67.27, 79.29) | 0.051 | |
|  |  | 61–80 | 73.72 (65.17, 80.17) | 58.13 (48.32, 66.09) | 0.191 | |
|  |  | >80 | 44.61 (13.26, 64.62) | 52.81 (33.84, 66.34) | 0.805 | |
|  | AZ-AZ-PZ | 18–40 | 79.45 (71.12, 85.38) | -6.17 (-37.30, 17.90) | <0.001 | |
|  |  | 41–60 | 89.04 (86.42, 91.15) | 70.09 (64.83, 74.56) | <0.001 | |
|  |  | 61–80 | 90.52 (89.22, 91.66) | 83.81 (82.06, 85.40) | <0.001 | |
|  |  | >80 | 81.17 (77.20, 84.45) | 63.82 (57.90, 68.91) | 0.003 | |
|  | AZ-AZ-MN | 18–40 | 92.78 (81.13, 97.24) | 34.53 (-30.81, 67.23) | 0.035 | |
|  |  | 41–60 | 92.41 (87.40, 95.43) | 76.86 (66.59, 83.98) | 0.089 | |
|  |  | 61–80 | 91.92 (89.53, 93.77) | 86.80 (83.98, 89.12) | 0.138 | |
|  |  | >80 | 80.72 (72.65, 86.41) | 47.41 (31.79, 59.45) | 0.011 | |
| *against fatal COVID-19* |  |  |  |  |  | |
|  | SV-SV-AZ | 18–40 | 81.76 (67.25, 89.84) | 68.09 (48.58, 80.20) | 0.121 | |
|  |  | 41–60 | 85.19 (80.26, 88.88) | 86.69 (83.21, 89.44) | 0.596 | |
|  |  | 61–80 | 95.22 (89.03, 97.92) | 96.26 (92.88, 98.04) | 0.723 | |
|  |  | >80 | 57.89 (12.73, 79.68) | 69.78 (46.89, 82.81) | 0.584 | |
|  | SV-AZ-AZ | 18–40 | 69.72 (39.71, 84.79) | 59.84 (32.57, 76.09) | 0.784 | |
|  |  | 41–60 | 76.57 (67.07, 83.33) | 78.45 (72.01, 83.42) | 0.877 | |
|  |  | 61–80 | 71.31 (62.70, 77.93) | 55.12 (45.20, 63.23) | 0.227 | |
|  |  | >80 | 67.18 (47.38, 79.52) | 31.22 (2.62, 51.42) | 0.257 | |
|  | SV-AZ-PZ | 18–40 | 75.26 (57.56, 85.58) | 83.56 (74.77, 89.29) | 0.646 | |
|  |  | 41–60 | 78.18 (72.07, 82.95) | 63.07 (55.27, 69.50) | 0.114 | |
|  |  | 61–80 | 81.83 (77.47, 85.34) | 66.60 (60.56, 71.71) | 0.033 | |
|  |  | >80 | 67.80 (54.74, 77.09) | 9.00 (-17.43, 29.48) | 0.017 | |
|  | SP-SP-PZ | 18–40 | 69.22 (49.41, 81.27) | 43.44 (17.32, 61.31) | 0.273 | |
|  |  | 41–60 | 84.24 (76.87, 89.26) | 82.17 (76.25, 86.61) | 0.804 | |
|  |  | 61–80 | 75.41 (63.58, 83.40) | 70.83 (60.87, 78.25) | 0.744 | |
|  |  | >80 | 48.96 (11.76, 70.48) | 64.99 (46.94, 76.90) | 0.652 | |
|  | AZ-AZ-PZ | 18–40 | 87.34 (76.74, 93.12) | 33.64 (-4.11, 57.70) | 0.021 | |
|  |  | 41–60 | 89.33 (85.77, 92.00) | 65.20 (56.75, 72.00) | <0.001 | |
|  |  | 61–80 | 90.98 (89.25, 92.44) | 88.03 (86.23, 89.60) | 0.194 | |
|  |  | >80 | 85.41 (81.28, 88.63) | 72.48 (66.58, 77.34) | 0.356 | |
|  | AZ-AZ-MN | 18–40 | 100.00 (NA, 100.00) | 100.00 (NA, 100.00) | 0.989 | |
|  |  | 41–60 | 94.61 (87.84, 97.61) | 83.70 (70.72, 90.93) | 0.285 | |
|  |  | 61–80 | 93.22 (90.10, 95.35) | 89.42 (86.00, 92.01) | 0.361 | |
|  |  | >80 | 89.43 (81.29, 94.04) | 86.96 (80.12, 91.44) | 0.783 | |

AZ, Astrazeneca (ChAdOx1); MN, Moderna (mRNA-1273); PZ, Pfizer/BioNTech (BNT162b2); SP, Sinopharm (BBIBP-CorV); SV, Sinovac (CoronaVac); VE, vaccine effectiveness
